# Variation in the feasibility and acceptability of electronic patient-reported outcome measures in patients with inflammatory arthritis

**DOI:** 10.1093/rap/rkag026

**Published:** 2026-02-17

**Authors:** Natasha Cox, Chelsea Kettle, Haoboo Wang, Shouma Dutta, Jon Packham, James Galloway, Jonathan Hill, Sara Muller, Samantha Hider, Zoe Paskins, Laurna Bullock, Ian C Scott

**Affiliations:** Centre for Musculoskeletal Health Research, School of Medicine, Keele University, Keele, UK; Haywood Academic Rheumatology Centre, Haywood Hospital, Midlands Partnership University NHS Foundation Trust, Staffordshire, UK; Centre for Musculoskeletal Health Research, School of Medicine, Keele University, Keele, UK; Haywood Academic Rheumatology Centre, Haywood Hospital, Midlands Partnership University NHS Foundation Trust, Staffordshire, UK; Haywood Academic Rheumatology Centre, Haywood Hospital, Midlands Partnership University NHS Foundation Trust, Staffordshire, UK; Centre for Musculoskeletal Health Research, School of Medicine, Keele University, Keele, UK; Haywood Academic Rheumatology Centre, Haywood Hospital, Midlands Partnership University NHS Foundation Trust, Staffordshire, UK; Centre for Rheumatic Disease, King’s College London, London, UK; Centre for Musculoskeletal Health Research, School of Medicine, Keele University, Keele, UK; Haywood Academic Rheumatology Centre, Haywood Hospital, Midlands Partnership University NHS Foundation Trust, Staffordshire, UK; Centre for Musculoskeletal Health Research, School of Medicine, Keele University, Keele, UK; Centre for Musculoskeletal Health Research, School of Medicine, Keele University, Keele, UK; Haywood Academic Rheumatology Centre, Haywood Hospital, Midlands Partnership University NHS Foundation Trust, Staffordshire, UK; Centre for Musculoskeletal Health Research, School of Medicine, Keele University, Keele, UK; Haywood Academic Rheumatology Centre, Haywood Hospital, Midlands Partnership University NHS Foundation Trust, Staffordshire, UK; Centre for Musculoskeletal Health Research, School of Medicine, Keele University, Keele, UK; Centre for Musculoskeletal Health Research, School of Medicine, Keele University, Keele, UK; Haywood Academic Rheumatology Centre, Haywood Hospital, Midlands Partnership University NHS Foundation Trust, Staffordshire, UK

**Keywords:** patient-reported outcome measures, rheumatoid arthritis, spondyloarthropathy, patient outcomes, mixed methods research

## Abstract

**Objectives:**

While electronic patient-reported outcome measures (ePROMs) can facilitate innovative, holistic care for patients with inflammatory arthritis (IA), their implementation could inadvertently worsen health inequalities. This mixed methods study aimed to evaluate their feasibility and acceptability in routine care and how this varied by factors potentially impacting digital inclusion.

**Methods:**

Patients with IA were invited to complete ePROMs before/at their appointment on a National Health Service digital platform (Haywood Arthritis Portal). A cross-sectional survey and semi-structured interviews were conducted in consenting patients and healthcare professionals (HCPs). Acceptability was evaluated using the Theoretical Framework of Acceptability. Survey responses were summarised descriptively. Statistical tests assessed global acceptability responses in relation to factors associated with digital inclusivity. Interviews were analysed using the Rigorous and Accelerated Data Reduction technique. Quantitative and qualitative findings were triangulated.

**Results:**

A total of 336 patients and 11 HCPs were surveyed; 12 patients and 5 HCPs were interviewed. Patient surveys/interviews demonstrated high ePROMs acceptability (89% found ePROMs completely acceptable/acceptable; 89% felt they benefitted care). Acceptability was lower in those who were older (*P* < 0.001), lacked internet access (*P* = 0.009) and had low general/e-health literacy/digital skills (*P* < 0.001). The largest differences were in those with *vs* without essential digital skills (93.0% *vs* 42.9% rating ePROMs acceptable/completely acceptable). Patient and HCP interviews also demonstrated inclusivity concerns. All HCPs considered ePROMs acceptable.

**Conclusion:**

While using ePROMs is feasible and highly acceptable to patients with IA and HCPs, acceptability is lower in patients who are older, less health literate and with lower digital skills/access. These factors require careful consideration in ePROMs implementation to avoid worsening health inequalities.

Key Messages

- ePROMs are acceptable to most patients with IA and to HCPs.
- Acceptability is lower in patients who are older, less health literate and with lower digital skills/access.
- Digital inclusion factors require careful consideration in ePROMs implementation to avoid worsening health inequalities.

## Introduction

Inflammatory arthritis (IA) is the most common group of conditions under National Health Service (NHS) rheumatology service follow-up. It affects >1% of adults in England, with far-reaching personal and societal impacts [1]. Ensuring patients with IA receive high-quality care, aligning treat-to-target strategies with biopsychosocial approaches, is therefore crucial. Patient-reported outcome measures (PROMs)—standardised, validated questionnaires establishing patients’ views on their health—are ideally placed to support this, with many available to assess disease activity, pain, function and quality of life. While their routine use is advocated in the Getting it Right the First Time (GIRFT) rheumatology report [2], using paper-based methods to collect and summarise PROMs to inform real-time care is infeasible, with electronic PROMs (ePROMs) required. It is anticipated that ePROMs can not only support holistic IA management but also enable care innovations like patient-initiated follow-up, aligning with current government policy to transform the NHS ‘from analogue to digital’ [3].

The crucial first step in ePROMs implementation is to ensure their feasibility and acceptability to patients and healthcare professionals (HCPs). To date, this has been examined in three studies in routine NHS IA care (Supplementary Table S1) [4–6]. While these suggested good acceptability for patients possessing the digital access/skills to complete ePROMs online or on a smartphone app, they did not consider patients who are unable to do this. Evaluating acceptability in this patient group is vital to ensure they are not left behind in a digitally evolving NHS, exacerbating health inequalities. Our study aimed to address this, evaluating the feasibility and acceptability of ePROMs in patients with IA and their treating HCPs, and exploring how this varies across various factors that could impact digital inclusion.

## Methods

### ePROMs system

The Haywood Arthritis Portal (HAP) collected ePROMs [7]. This web-based, NHS platform was co-designed by the Midlands Partnership University NHS Foundation Trust (MPFT) rheumatology and digital teams in collaboration with service users. Patients under the care of MPFT rheumatology can complete ePROMs on their smartphone, tablet or computer. These data are fed-forward into their electronic health record, displayed on a summary dashboard that can be rapidly reviewed by their HCP before the patient has entered the room and integrated with other data (i.e. joint counts to calculate the 28-joint DAS [DAS28]). Patients attending dedicated IA clinics with a registered mobile phone number receive a text message prompt to complete HAP entries 1 week pre-appointment. People not completing them can complete ePROMs on digital tablets at their appointment (with healthcare support worker assistance, if needed).

### Study design

An explanatory, sequential, mixed methods study was conducted, involving a cross-sectional survey and semi-structured interviews in patients with IA and their HCPs.

### Underpinning theory

**Figure 1:**
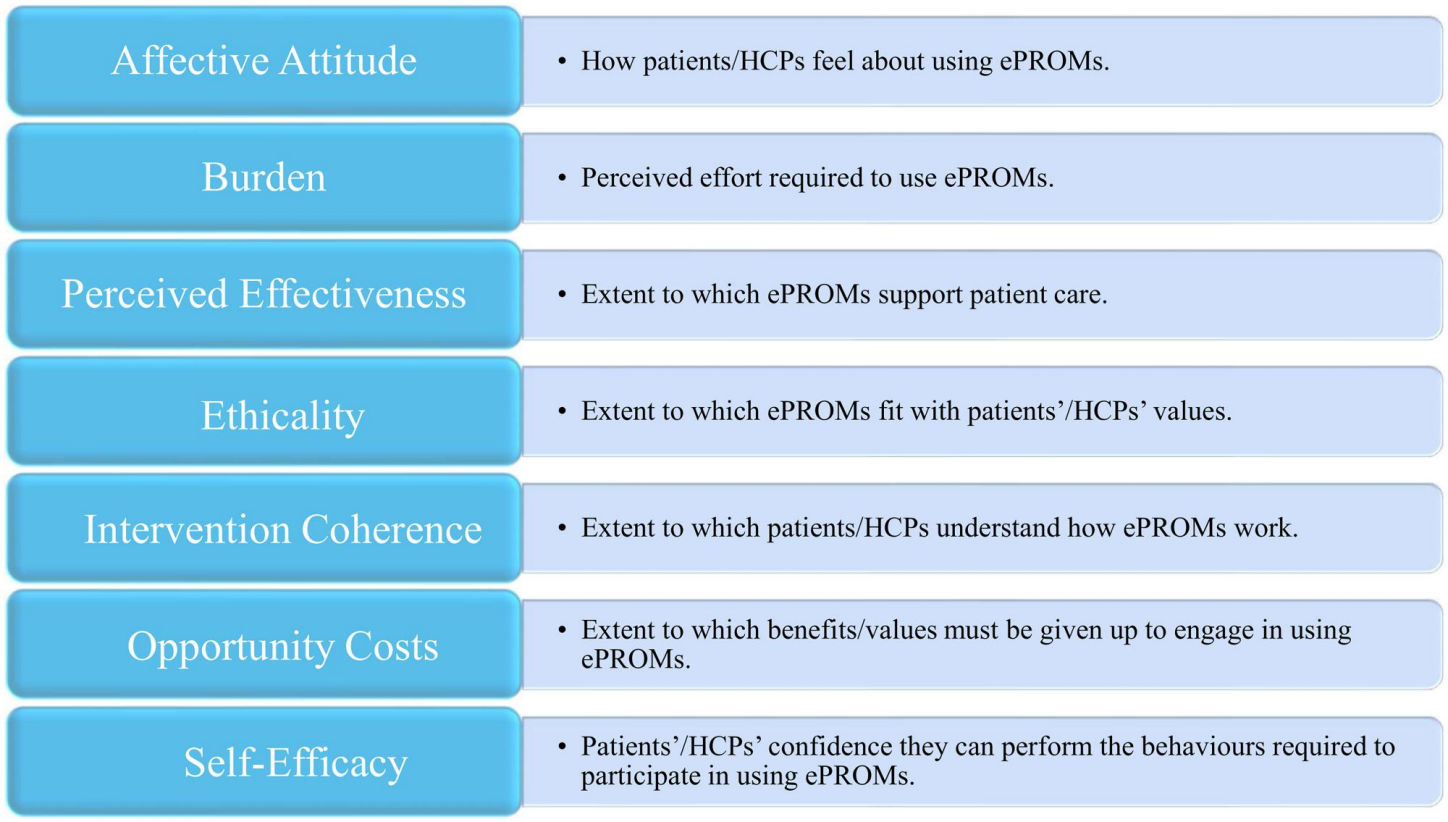
Overview of the seven component constructs of the TFA in the context of this study

This study is underpinned by Normalisation Process Theory (NPT) [8], which supports understanding of the collaborative ‘work’ needed for interventions to be implemented, embedded and normalised in routine care, and the Theoretical Framework of Acceptability (TFA) [9], examining acceptability against seven constructs comprising affective attitude, burden, perceived effectiveness, ethicality, intervention coherence, opportunity costs and self-efficacy (Fig. 1).

### Patient and public involvement and engagement (PPIE)

A PPIE group, comprising two people with lived experience, reviewed patient-facing documentation and interview topic guides, advising on the phrasing of questions to ensure clarity and understanding and identifying any gaps in their content from a patient perspective. This led to several changes, such as the inclusion of an option of ‘I did not feel confident completing it at home without support from my healthcare team’ in the clinic-completer survey question about why the portal was not completed at home.

### Survey

#### Participants

Patient participants comprised adults with RA, peripheral or axial SpA or undifferentiated IA as recorded in their electronic health record who were invited to complete the HAP and consented to the survey. HCP participants comprised those undertaking IA consultations, viewing HAP ePROMs data in ≥20 consultations, who provided consent. Participants could complete one survey, with no further exclusion criteria.

#### Recruitment

Eligible patients were approached by their direct care team at their appointment. Consenting participants could complete the survey on tablets pr paper immediately post-appointment or at home (returning paper surveys using prepaid envelopes). Eligible HCPs were approached by a researcher, completing the survey online.

#### Content

This differed for patients according to whether they completed the HAP at home (home completers), in clinic (clinic completers) or declined completion (non-completers) (Supplementary Data S1–S3). All surveys evaluated internet access (bespoke questions), internet use (Office for National Statistics categories) [10], general health literacy (single-item screening question) [11, 12], eHealth literacy (eHealth Literacy Scale) [13], digital skills to safely benefit from the digital world [14, 15] (people are ‘digitally excluded’ if they lack any skills, have ‘partial foundation’ if they have some skills and are ‘foundation’ level if they have all skills), ethnicity (Office for National Statistics categories) and postcode (for Index of Multiple Deprivation [IMD] scores). Home/clinic completer surveys also evaluated ePROMs acceptability using the generic TFA eight-item questionnaire (examining acceptability against the seven constructs [Fig. 1] alongside their perspective of global acceptability) [9], HAP usability (health information technology assessment scale) [16] and use of ePROMs responses by their HCP (bespoke questions). Non-completer surveys also evaluated non-completion reasons. Age, gender and IA type were captured from NHS records.

HCP surveys (Supplementary Data S4) evaluated ePROMs acceptability, implementation and usability using established instruments (generic TFA; 23-item Normalisation Measures Development [NoMAD] instrument [17]; Health Information Technology Usability Evaluation Scale [16]) and bespoke questions (relating to how they used ePROMs in the clinic and which ePROMs they considered helpful).

### Semi-structured interviews

#### Participants

Patient and HCP survey completers who provided consent and had access to telephone/video calling services were eligible. HCPs who were research team members were excluded. As with the survey, participants could participate in one interview, with no further exclusion criteria applied.

#### Recruitment

Survey completers who provided consent-to-contact were approached by a researcher, consented and an interview arranged (via telephone/video call for patients or telephone/video call/in-person for HCPs). Patients were purposively sampled to capture variation in age, gender and IA type (hypothesised to potentially impact ePROMs acceptability) and global acceptability levels.

#### Content

Topic guides (Supplementary Data S5–S7) were informed by TFA constructs [9], with NPT also informing HCP topic guides. These were iteratively updated, based on early findings.

### Analysis

Descriptive statistics summarised survey responses as proportions or means (with 95% CIs). Patient responses for each TFA domain were summarised overall and by completion type (home, clinic). Chi-squared tests (or Fisher’s exact tests for expected cell counts <5) compared differences in patient TFA global acceptability responses by age, gender, IA type, IMD quintiles, internet access, smartphone ownership, health literacy, eHealth literacy and digital skill categories (ethnicity was not considered, as most were of ‘White’ ethnicity). Analysis of variance compared global acceptability responses by age. Free-text reasons for non-completion were analysed descriptively, although, as only seven patients declined HAP completion (six providing comments), their views may not represent all non-completers. Data were analysed using Stata version 18 (StataCorp, College Station, TX, USA) and R version 4.3.1 (R Foundation for Statistical Computing, Vienna, Austria).

The Rigorous and Accelerated Data Reduction technique analysed interviews using a team approach (five researchers) for data familiarisation, organisation, reduction and inductive coding [18]. Data were deductively mapped against TFA domains through regular group discussion using Excel (Microsoft, Redmond, WA, USA). A side-by-side joint display integrated quantitative and qualitative findings to generate deeper insights [19].

### Ethical approval

Approval was obtained from the London Bridge Research Ethics Committee (reference 22/PR/1282). All participants were given a patient information sheet and provided informed consent prior to surveys and interviews.

## Results

### Patient survey

#### Patient characteristics

Of 373 eligible people approached, 336 (90.1%) consented and completed the survey. Most were female (70.7%) and had RA (65.9%) (Table 1). The sample lacked ethnic diversity, with 98.8% being of White ethnicity (and recruited from a single centre), potentially limiting generalisability of the findings to more ethnically diverse populations. The mean age was 59.9 years (95% CI 58.5, 61.3). Many were retired (43.4%), one-quarter were in the most deprived IMD quintile and one-fifth had low health literacy. While most (91.6%) could access the internet, fewer (79.2%) had their own smartphone. Approximately one-fifth lacked essential digital skills to access the online world (21.6% partial foundation or digitally excluded); one-quarter (24.1%) had low eHealth literacy.

**Table 1.** Patient characteristics, digital access and skills.

Characteristics		Survey participants	Interview participants
Arthritis type and sociodemographic characteristic			
IA type, *n* (%)	RA	220 (65.9)	8 (72.7)
	Peripheral PsA	32 (9.6)	1 (9.1)
	Axial SpA	52 (15.6)	0 (0)
	Other^a^	30 (9)	2 (18.2)
Age, years, mean (95% CI)		59.9 (58.5, 61.3)	61.1 (51.8, 70.4)
Gender, *n* (%)	Female	236 (70.7)	7 (63.6)
Ethnicity, *n* (%)	White	316 (98.8)	11 (100)
	Non-white	4 (1.3)	0 (0)
Occupation, *n* (%)	Employed	118 (37.1)	5 (45.5)
	Unemployed	54 (17.0)	2 (18.2)
	Retired	138 (43.4)	4 (36.4)
	Other	8 (2.5)	0 (0)
IMD quintiles, *n* (%)	1 (most deprived)	79 (25.1)	2 (20)
	2	47 (14.9)	1 (10)
	3	56 (17.8)	1 (10)
	4	69 (21.9)	3 (30)
	5 (least deprived)	64 (20.3)	3 (30)
Low health literacy, *n* (%)		65 (20.5)	2 (18.2)
Digital access and skills			
Access to internet, *n* (%)		295 (91.6)	10 (90.9)
Access to internet on own smartphone, *n* (%)		240 (79.2)	9 (81.8)
Digital skills, *n* (%)	Foundation	247 (78.4)	9 (81.8)
	Partial foundation	52 (16.5)	2 (18.2)
	Digitally excluded	16 (5.1)	0 (0)
Low eHealth literacy, *n* (%)		76 (24.1)	3 (27.3)

a Includes peripheral SpA other than PsA, and undifferentiated IA.

A total of 172 (51.2%) patients completed the HAP at home, 157 (46.7%) in the clinic and 7 (2.1%) declined to complete it. Some differences in characteristics across these groups were seen (Supplementary Table S2), with higher proportions of clinic completers and non-completers having low health/eHealth literacy when compared with home completers. Conversely, higher proportions of home completers owned a smartphone and/or had foundation digital skills.

#### ePROMs acceptability

**Figure 2:**
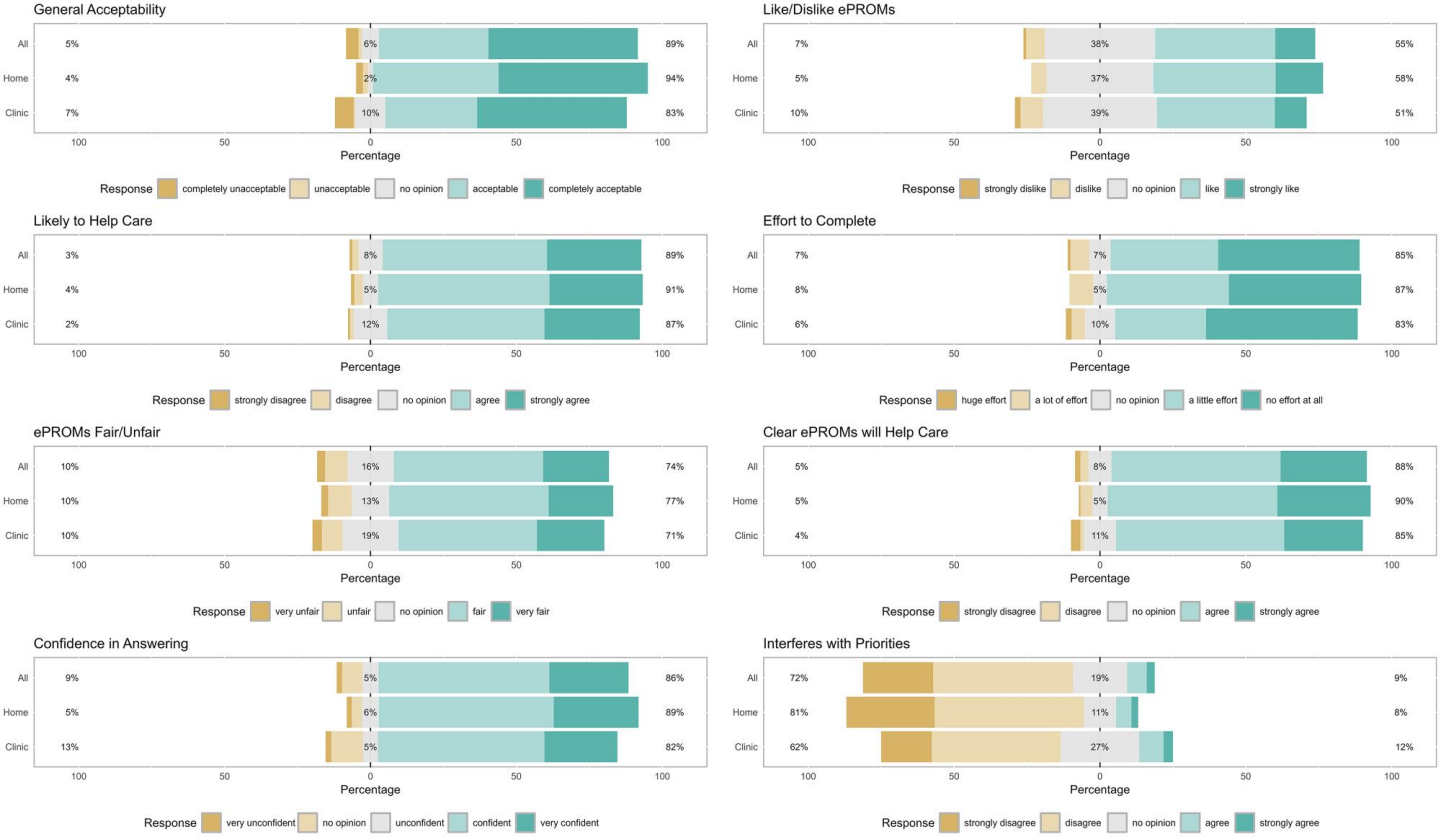
Theoretical framework of acceptability global and component construct responses by portal completion type. Home: people completing the HAP at home on their own device; clinic: people completing the HAP in the clinic on NHS digital tablets; all: both home and clinic completers; percentage on the left of the graph represents those selecting the two Likert-type response options furthest to the left of the relevant legend (e.g. for general acceptability, those selecting ‘completely unacceptable’ or ‘unacceptable’); percentage in the middle of the graph represents those selecting the middle Likert-type response option (e.g. for general acceptability, the percentage selecting ‘no opinion’); percentage on the right of the graph represents those selecting the two Likert-type response options furthest to the right (e.g. for general acceptability, those selecting ‘acceptable’ or ‘completely acceptable’)

Acceptability levels were high (Table 2 and Fig. 2), as 88.7% considered ePROMs acceptable/completely acceptable and 54.9% liked/strongly liked ePROMs. Most felt completing ePROMs was fair/very fair (73.8%), agreed/strongly agreed they were likely to help their care (88.7%), were confident/very confident in answering ePROMs (85.7%), agreed/strongly agreed they understood how ePROMs helped their care (87.5%) and thought completing them required no/little effort (85.2%).

**Table 2:** Joint display of patient quantitative and qualitative findings on ePROMs acceptability.

TFA domain	Quantitative findings, *n* (%)	Qualitative findings	Meta-inference
Global acceptability	Acceptable/completely acceptable: 291 (88.7) No opinion: 19 (5.8) Unacceptable/completely unacceptable: 18 (5.5)	–	–
Affective attitude (like/dislike ePROMs)	Like/strongly like: 180 (54.9) No opinion: 124 (37.8) Dislike/strongly dislike: 24 (7.3)	Overall, patients liked or were neutral about using ePROMs. Example quote: ‘I can just remember saying to my mum, “Oh, I’m ever so glad they’ve asked these questions”’. (patient_1066)	Convergence: most liked or had no opinion about completing ePROMs. Reasons for this are demonstrated in other domains.
Ethicality (fairness of ePROMs)	Fair/very fair: 242 (73.8) No opinion: 52 (15.9) Unfair/very unfair: 34 (10.4)	Patients often thought ePROMs were fair and everybody should be offered the opportunity to complete them. However, challenges regarding inclusivity hindered fairness, and sometimes, support was required. Example quote: ‘My granddaughter was here to help me log in and that ‘cos I’m not very technically minded’. (patient_5008) Patients recognised efforts to minimise inequalities, e.g. multiple ePROMs completion methods and in-clinic support from staff. Example quote: ‘…providing they have got access to the internet…the older generation, have they got access to it? But then if they don’t answer them online at home, provided that they’ve got the opportunity to answer them in the clinic, then it’s all good’. (patient_1066)	Convergence: most thought ePROMs were fair/very fair, providing the option for people with arthritis to record their health information, if they wish to. Few thought ePROMs were unfair/very unfair, potentially due to some having difficulty completing them at home.
Perceived effectiveness (ePROMs likely to help care)	Agree/strongly agree: 291 (88.7) No opinion: 27 (8.2) Disagree/strongly disagree: 10 (3.0)	The majority thought ePROMs had been or could be effective in helping their arthritis care. Example quote: ‘It probably makes you think about how you actually are before you get there if you like, to fill the portal in, so you’ve already sort of got the answer to the questions in your head that you might be asked when you get there’. (patient_2055) Many patients recognised the value of ePROMs and suggested opportunities for wider implementation developments to enhance effectiveness further, such as to triage waiting lists. Example quote: ‘I think for people who you know, are in a reasonable situation and don’t need to be seen too often, maybe something could be set up where you get an appointment without seeing a consultant or a nurse; you’re just asked to fill in the portal, and that in effect is your appointment. You’re just giving them the information, and then if they see anything that they need to discuss, they’ll get in touch or make you an appointment’. (patient_2055)	Convergence: most perceived ePROMs as likely to help care because of perceived or experienced benefits. Many potential wider implementation suggestions were made, highlighting the perceived effectiveness patients felt towards ePROMs.
Self-efficacy (confident answering ePROMs)	Confident/very confident: 281 (85.7) No opinion: 23 (7.0) Unconfident/very unconfident: 24 (7.3)	Most were confident, although less so when answering retrospective questions. Patients thought familiarity over time would support increased confidence. Example quote: ‘When they sort of ask you how have you been feeling in the last fortnight, I have to think, “well I don’t know really”…I felt that was a little bit difficult to answer in some ways’. (patient_5008)	Convergence: most reported high self-efficacy when answering ePROMs, because they were clearly presented and easy to complete. The minority with low confidence completing ePROMs may have this due to lower digital skills, or due to problems answering retrospective questions.
Intervention coherence (clear how ePROMs help care)	Agree/strongly agree: 287 (87.5) No opinion: 26 (7.9) Disagree/strongly disagree: 15 (4.6)	Patients understood how ePROMs helped care, how they worked and were willing to use them. Example quote: ‘it’s information that your consultant needs to know [mhmm]. You can pass it on to them straight through…If you’ve got the tools to make things easier, make it easier’. (patient_1055)	Convergence: most agreed/strongly agreed that they understood how ePROMs help care, with many providing examples of how ePROMs ‘made sense’ as a helpful component of their care.
Burden (effort to complete ePROMs)	No effort/little effort: 278 (85.2) No opinion: 24 (7.4) A lot of effort/huge effort: 24 (7.4)	Although patients reflected that ePROMs required minimal effort and did not interfere with other priorities, many also described costs and burdens to engaging with ePROMs (e.g. completing questions when no changes present since last entry). Example quote: ‘I’ve got just as busy a life as the doctors and the nurses. So, if it’s going to make my life simpler and their lives simpler, then I’m quite happy to do it…As long as it didn’t stop me seeing [clinician] once a year and my nurses’. (patient_1092)	Convergence: most thought that ePROMs required no/little effort and disagreed/strongly disagreed that they interfered with other priorities. Reasons for this were that completion could be done leisurely at home or while waiting in the clinic.
Opportunity costs (ePROMs interfere with other priorities)	Agree/strongly agree: 31 (9.5) No opinion: 61 (18.6) Disagree/strongly disagree: 236 (72.0)		

Home completers (on average were younger, more digitally skilled, with higher health literacy/eHealth literacy) viewed ePROMs as more acceptable across all TFA domains than clinic completers (Fig. 2, Supplementary Table S2). Although differences were small, home completers consistently reported greater acceptability and lower perceived burden. The only TFA domain in which large differences were seen was ‘opportunity costs’, with 81.4% of home completers *vs* 61.5% of clinic completers disagreeing/strongly disagreeing that ePROMs interfered with other priorities.

**Figure 3:**
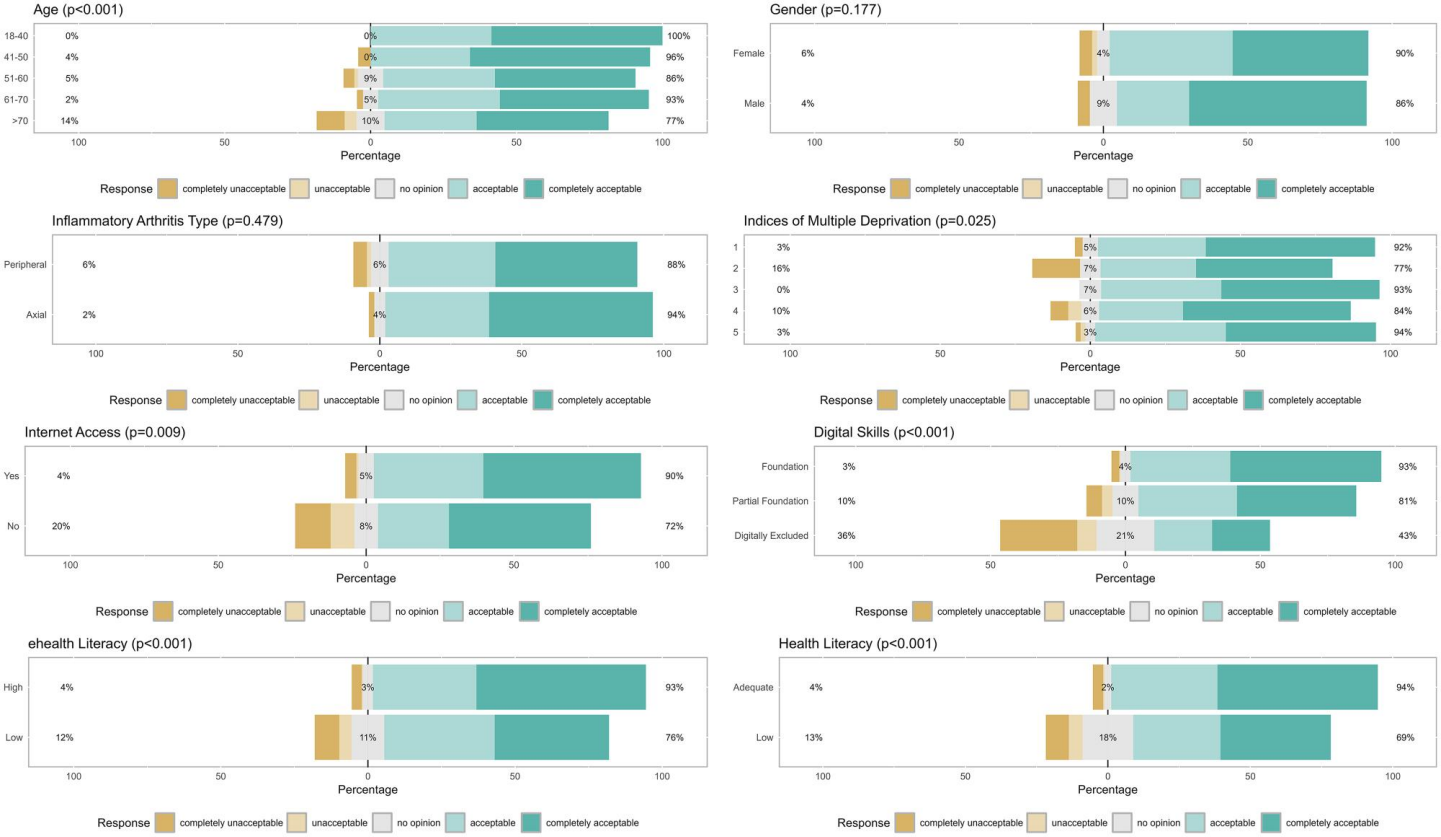
Variation in global acceptability responses by patient factors. Percentage on the left of the graph represents those selecting the Likert-type response options ‘completely unacceptable’ or ‘unacceptable’; percentage in the middle of the graph represents those selecting the Likert-type response option ‘no opinion’; percentage on the right of the graph represents those selecting the Likert-type response options ‘acceptable’ or ‘completely acceptable’. *P*-values are from chi-squared tests (or Fisher’s exact tests for expected cell counts <5) except for age (in which differences in global acceptability responses were examined using analysis of variance)

Global acceptability levels differed by various factors (Fig. 3). A statistically significant difference in global acceptability was observed by age (*P* < 0.001), with those rating ePROMs as acceptable/completely acceptable being, on average, 9 years younger (mean 58.8 years [95% CI 57.3, 60.3]) than those having no opinion (67.4 years [95% CI 63.1, 71.7]) or rating them unacceptable/completely unacceptable (67.8 years [95% CI 62.7, 73.0]). Significant differences in global acceptability were also observed by internet access (90.3% with *vs* 72.0% without access rated ePROMs acceptable/completely acceptable, representing a difference of 18.3%; *P* = 0.009), general health literacy (93.5% with high *vs* 69.4% with low health literacy rated them acceptable/completely acceptable, representing a difference of 24.1%; *P* < 0.001) and eHealth literacy (92.8% with high *vs* 76.4% with low eHealth literacy rated them acceptable/completely acceptable, representing a difference of 16.4%; *P* < 0.001). The largest difference in global acceptability was seen by digital skill levels (*P* < 0.001); 93.0%, 80.8% and 42.9% with foundation skills, partial foundation skills and those digitally excluded considering ePROMs acceptable/completely acceptable, respectively (representing a difference of 50.1% between those with foundation skills and those who were classified as digitally excluded). While global acceptability scores differed across IMD quintiles (*P* = 0.025), no clear trend was observed. Acceptability did not differ by gender (*P* = 0.18) or IA type (*P* = 0.48). Similar trends in global acceptability levels were seen in home and clinic completers (Supplementary Figs S1 and S2), although these changes were only statistically significant for age (*P* = 0.020) and general health literacy (*P* = 0.011) in home completers and age (*P* = 0.048), general health literacy (*P* < 0.001) and digital skill levels (*P* < 0.001) in clinic completers.

#### HAP usability, perceived use of ePROMs by HCPs and perceived impact on care

Most (79.6%) agreed/strongly agreed the HAP was easy to use. A substantial minority felt their ePROMs data were not looked at or discussed with them during their consultation, which has important implications for implementation. Specifically, 10.8%, 25.9% and 18.4% of respondents disagreed/strongly disagreed with statements their HCP had looked at their ePROMs data, discussed their answers with them and used them to inform their care. However, up to two-thirds felt ePROMs benefited their appointment, with 68.7% and 55.1% agreeing/strongly agreeing that the questions helped them think about the problems they needed to discuss and led to a better appointment.

#### Non-completion reasons

Free-text responses for non-completion were available in six (of seven) patients. Two reported this was due to unfamiliarity with digital systems (‘At the age of 80 I cannot use tablets or computers’; ‘I am not familiar with modern technology and only use my mobile telephone in emergencies’), one reported a technical problem with the HAP, one reported a lack of time, one reported they did not read the communication about it correctly and another reported uncertainty about the HAP’s purpose.

### HCP survey

#### HCP characteristics

The 11 HCP survey completers were from a range of backgrounds (consultants, specialist registrars, extended scope physiotherapists, clinical nurse specialists) and were mostly between 30 and 59 years of age, female (72.7%) and of White ethnicity (72.7%).

#### Acceptability

Acceptability was high. All HCPs rated ePROMs as acceptable/completely acceptable, liked/strongly liked having ePROMs data and felt confident/very confident using ePROMs in routine care (Table 3). Most agreed/strongly agreed that ePROMs were likely to improve patient care (90.9%), were clear how ePROMs help care (90.9%) and felt it was no/little effort to use ePROMs (90.9%). Fewer were positive about opportunity costs (approximately two-thirds [63.6%] disagreeing/strongly disagreeing that using ePROMs interfered with other priorities) and about ePROMs fairness, with responses similar across fairness categories (36.4% unfair/very unfair, 36.4% no opinion, 27.3% fair/very fair).

**Table 3:** Joint display of healthcare professional quantitative and qualitative findings on ePROMs acceptability.

TFA domain	Quantitative findings, *n* (%)	Qualitative findings	Meta-inference
Global acceptability	Acceptable/completely acceptable: 11 (100) No opinion: 0 (0) Unacceptable/completely unacceptable: 0 (0)	–	–
Affective attitude (like/dislike ePROMs)	Like/strongly like: 11 (100) No opinion: 0 (0) Dislike/strongly dislike: 0 (0)	All expressed positive attitudes towards using ePROMs. They were described as a useful tool that benefitted day-to-day practice.	Convergence: all liked/strongly liked ePROMs, with underlying reasons demonstrated in other domains.
Ethicality (fairness of ePROMs)	Fair/very fair: 3 (27.3) No opinion: 4 (36.4) Unfair/very unfair: 4 (36.4)	HCPs had mixed views on ePROMs fairness. They felt it was fair to offer all patients the opportunity to complete ePROMs but described many challenges to being inclusive, such as limited options for non-readers, people that don’t speak English, those with low digital access/skills and limited physical dexterity. Example quote: ‘I guess one thing is already being done and that’s giving people the opportunity to complete it…in the waiting room before they come in’. (HCP_4008)	Convergence: mixed HCP responses to fairness of ePROMs; likely due to the commonly described view that while it was fair to offer patients the opportunity to complete ePROMs, this was compromised by inclusivity concerns.
Perceived effectiveness (ePROMs likely to help care)	Agree/strongly agree: 10 (90.9) No opinion: 1 (9.1) Disagree/strongly disagree: 0 (0)	Most thought ePROMs had been effective or had the potential to be effective in improving the care of people with IA. Example quote: ‘If you wanted to discuss escalation or de-escalation of care, by having these sorts of facts and figures and a graph in front of you, you can encourage the patient to take better decisions’. (HCP_4007) Example quote: ‘If you imagine a scenario of stable ankylosing spondylitis patients who don’t need to come into the clinic regularly but are able to feed their ePROMs data in and that there’s somebody managing this then they are not offered needless appointments’. (HCP_4007)	Convergence: most agreed/strongly agreed that ePROMs were likely to be effective due to reflexive monitoring of benefits experienced such as helping to inform treatment decisions and conversations in consultations. Many HCPs also proposed future implementation developments to enhance effectiveness further.
Self-efficacy (confident using ePROMs)	Confident/very confident: 11 (100) No opinion: 0 (0) Unconfident/very unconfident: 0 (0)	Most felt confident engaging with ePROMs and in the validity of ePROMs. Example quote: ‘I feel absolute confidence in using them. There are guidelines and treatment decisions that can be made based on these scores very easily’. (HCP_4007) However, one HCP reported that they initially lacked confidence in using ePROMs due to lack of instruction/training. Example quote: ‘It is a bit tricky initially, because I don’t know if there’s, maybe I missed it, but I don’t think there was any kind of training or anything like that…So I relied on other people showing me’. (HCP_4002) Some HCPs felt that their confidence in using and interpreting ePROMs would increase with continued implementation. Example quote: ‘I’m a bit of an old-fashioned beast in some ways. You know, I trained in rheumatology a long time ago. Again, maybe it’s just familiarity and confidence in the ability to interpret them’. (HCP_4008) Despite this, some questioned patient confidence in using ePROMs due to misaligned responses compared with verbal reports in consultations. Example quote: ‘I think some of the patients, you know, the answers that they’ve put, don’t reflect what they tell you in the clinic. And sometimes I think they’ve just gone through it and kind of ticked, you know, ‘yes, yes, yes’, or whatever, rather than thought deeply about the questions’. (HCP_4002)	Convergence: most felt confident/very confident using ePROMs, mostly due to the system’s interface being described as user-friendly. However, some HCPs recognised opportunities to improve confidence further.
Intervention coherence (clear how ePROMs help care)	Agree/strongly agree: 10 (90.9) No opinion: 1 (9.1) Disagree/strongly disagree: 0 (0)	HCPs understood the purpose of integrating ePROMs into routine care but had some uncertainties regarding interpretation and how to manage ePROMs outputs, such as psychological problems. Example quote: ‘I think it would be helpful to have a more streamlined process for managing some of the issues identified, e.g. depression. I am not an expert in this and sometimes I feel uncomfortable asking GPs to deal with things when they are already swamped’. (HCP_4002)	Convergence: most agreed/strongly agreed that it was clear how ePROMs help care due to their experience of using them in practice. However, understanding of ePROMs could be strengthened according to HCPs.
Burden (effort to complete ePROMs)	No effort/little effort: 10 (90.9) No opinion: 0 (0) A lot of effort/huge effort: 1 (9.1)	HCPs reported there were minimal burdens/costs to engaging with ePROMs, and in some cases they saved time in clinic, providing easier access and comparison to historic patient health data and automatic calculation of disease activity scores. Example quote: ‘When I’m calculating a disease activity score, I can enter my clinician-assessed tender and swollen joint count and the blood test…combine it with the patient global and therefore that gives me the disease activity score. And obviously if the patient hasn’t recorded it in advance, then I’d have to ask the patient global question. So it avoids me having to do that’. (HCP_4008) Some HCPs acknowledged the individual work required to implement ePROMs, for example, having to use another system and re-enter data when the webpage timed out. However, most felt that this work was justified or could be minimised by future developments. Example quote: ‘The old-fashioned paper ones—you know, the question then is what you do with that piece of paper…file it into notes or would we have to take a photo of it and scan it in and things like that? So, and then it’s harder to find again’. (HCP_4013) One HCP recognised the need for collective action in implementing ePROMs across the clinical team, given the current mixed levels of cognitive participation in its use (due to preference for traditional methods). Example quote: ‘Whether there’ll be a problem within the team with implementing it, in terms of getting the whole clinical team on board…that might be a bit more nuanced’. (HCP_4002)	Convergence: most thought ePROMs required no/little effort and often did not interfere with other priorities. This appeared to be because ePROMs took little time and/or effort from clinic activities and in some cases saved time by alleviating pre-existing burdens from usual practice. Despite this, individual and collective work is required to implement ePROMs into routine practice.
Opportunity costs (ePROMs interfere with other priorities)	Agree/strongly agree: 2 (18.2) No opinion: 2 (18.2) Disagree/strongly disagree: 7 (63.6)		

#### Reported implementation of ePROMs

While 91.1% of HCPs felt they looked at ePROMs entries always/often, only 54.5% stated they discussed these with patients always/often. NoMAD responses demonstrated strong conceptual engagement, with scores generally high for items related to sense making (coherence) and cognitive participation (Supplementary Table S3; 90.9% agreed/strongly agreed they ‘can see the potential value of ePROMs’; 100% agreed/strongly agreed they were receptive to continuing to support ‘the use of ePROMs at our NHS trust’ and ‘working with colleagues in new ways to use ePROMs’), with the exception of staff having ‘a shared understanding of the purpose of ePROMs’ (63.6% agreed/strongly agreed). However, there was less perceived practical support for implementation, with scores lower in the domain of collective action, with 36.4% and 54.5% agreeing/strongly agreeing that ‘sufficient resources are available to support ePROMs’ and ‘sufficient training is provided to enable staff to use ePROMs’, respectively. Scores for reflexive monitoring were mixed (Supplementary Table S3).

#### Semi-structured interviews

Interviews were conducted remotely by a qualitative researcher (C.K., female) with 12 patients who were not known to the researcher (1 patient interview was unanalysable due to audio-recording failure) and 5 HCPs. Interviews lasted ≈30 min. A narrative summary of interview findings for each TFA domain are provided (supporting quotes in Tables 2 and 3 and Supplementary Tables S4 and S5).

#### Affective attitude

Both patients and HCPs generally liked ePROMs and were willing to use them.

#### Ethicality

Patients mostly considered ePROMs fair, believing everyone with IA should have access. Patients and HCPs recognised inclusivity challenges to ePROMs completion for patients (e.g. poor dexterity, limited digital access, low literacy) and some patients reported requiring support in completing ePROMs from relatives or staff. However, patients and HCPs thought the current approach—offering multiple completion methods and in-clinic staff support—increased fairness. HCPs thought that ePROMs supported good-quality care, although some reflected that inconsistent or non-use of ePROMs by colleagues could hinder care (e.g. omissions in clinical records), with decision-making implications.

#### Perceived effectiveness

Patients reported that ePROMs provided deeper health insights, improved recall of health information and alleviated anxieties of face-to-face discussions. HCPs felt they helped to inform treatment decisions, track health changes, assess disease activity and guide discussions. They also gathered information that might not be routinely covered (e.g. psychological well-being), enabling holistic care (although this could lengthen consultations). A key motivator to HCPs using ePROMs was championing by colleagues. While some HCPs were sceptical about moving away from traditional assessment methods, they could reflect on ePROMs’ benefits.

#### Self-efficacy

The HAP interface gave patients confidence in completing ePROMs, which increased with familiarity over time. However, limited recall (e.g. remembering symptoms over a 2-week period) and uncertainty in responding to some ePROM items (e.g. providing an ‘average’ score) meant some patients had difficulty answering retrospective questions. Other patients reported lower confidence due to limited digital skills. HCPs occasionally perceived misalignment between ePROMs and discussions, highlighting potential patient uncertainty in responding. Most HCPs trusted ePROMs’ validity, positively impacting their practice by informing treatment decisions.

#### Intervention coherence

Patients generally understood the purpose of ePROMs, being willing to complete them more frequently. Some reported initial uncertainty, gaining clarity after further exploration. Data security was often mentioned, with patients trusting their data were safe due to the information provided (e.g. NHS branding). HCPs shared an understanding of what ePROMs aimed to achieve, reflecting how their availability differed from usual care (e.g. encouraging conversations about non-routine topics, such as psychological well-being). Some HCPs reported that more training would help them understand how best to integrate ePROMs into routine practice. Both patients and HCPs recognised opportunities to enhance ePROMs use (e.g. triaging waiting lists, supporting patient-initiated follow-up), demonstrating a shared understanding of the value of ePROMs.

#### Burden and opportunity costs

Discussion of issues relating to these domains were closely aligned. Patients found completing ePROMs involved little effort, did not interfere with other priorities and took up no extra time when completed in the clinic. However, they stated that ePROMs completion should complement, not replace, usual care. Perceived burden and opportunity costs directly influenced implementation. HCPs expected ePROMs to be burdensome; however, some perceived they saved time (e.g. in calculating DASs). However, patients and HCPs commonly felt they repeated information captured in ePROMs in consultations, duplicating work. This caused doubt among patients regarding whether HCPs had viewed ePROM entries, with acknowledgement of completion advocated. Completing ePROMs in the clinic sometimes caused delays, requiring support from staff who were drawn from other responsibilities.

## Discussion

This mixed methods study has evaluated the feasibility and acceptability of ePROMs in the routine care of patients with IA and explored how these vary by factors that could affect digital inclusivity and exacerbate health inequalities. It focused on evaluating the feasibility and acceptability of ePROMs delivery, as opposed to ePROMs validity or disease-specific measurement performance. There were three key findings. First, it demonstrates that using ePROMs is acceptable to most patients and HCPs, with patients considering that all people with IA should have access to them. Second, it shows that acceptability levels are lower in people who are older, have low general health/eHealth literacy, lack internet access and have limited digital skills. Third, it highlights the importance of training and collaborative NHS teamwork when implementing ePROMs, with many HCPs identifying these as implementation barriers. Taken together, these findings support national GIRFT recommendations to drive forward routine PROMs capture in rheumatology departments to improve care, alongside the UK government’s strategy to enhance digital NHS care, with the caveat that steps are needed to ensure inclusivity. While we focused on using ePROMs in patients with IA (as they represent the most common group of conditions under long-term rheumatology follow-up and have a range of well-validated PROMs for numerous health aspects), our findings on ePROMs feasibility/acceptability are likely to be applicable to other long-term conditions, if they were to be implemented in the same way.

Our study replicates other NHS-based studies examining routine ePROMs use in patients with IA, which demonstrated good acceptability levels. However, our study additionally included patients not completing ePROMs on their own devices and explored factors that could affect acceptability. We found that ePROMs acceptability was lower in those who are older, lack internet access and have lower health literacy and digital skills. This latter factor was of particular importance, associating with the largest differences in global acceptability (>90% of those with digital foundation status *vs* <50% of those who were digitally excluded considered ePROMs acceptable/completely acceptable). It is widely acknowledged that digital exclusion can compound health inequalities, exacerbating challenges in accessing healthcare and resources to lead healthy lives [20]. Approximately 1.6 million people in the UK live offline and one-quarter of the population have the lowest levels of digital capability [21]. For the benefits of ePROMs in rheumatology departments to be fully realised, it is vital that consideration is given to ensuring digital inclusivity, adhering to principles outlined in the NHS framework for action on digital inclusion [20]. This issue is of topical importance, with the 10 Year Health Plan for England planning to harness the digital revolution to place power in the hands of patients and optimise service efficiencies. One example of this is the planned replacement of two-thirds of outpatient appointments with automated information, digital advice, specialist input and patient-initiated follow-up via the NHS app [22]. Our study indicates that careful consideration is needed to ensure the inclusion of people with limited digital skills in this new healthcare model.

Notably, approximately one-quarter of patients disagreed that HCPs discussed their ePROMs data with them, with a minority (11%) disagreeing that their HCP had looked at their ePROMs. This finding was echoed in interviews, with patients reporting HCPs repeated questions covered in ePROMs, raising doubts that data had been viewed. Of the surveyed HCPs, while 91% reported they looked at ePROMs ‘always/often’ and ‘strongly agreed/agreed’ they used ePROMs to make decisions, only 45% reported discussing ePROMs entries with patients ‘always/often’. Therefore, while HCPs were positive about having ePROMs data and interviews highlighted the benefits of discussing ePROMs results during consultations to support patients in making informed decisions about treatment changes, many did not place weight on discussing entries with patients. The same was observed in a survey of 119 rheumatologists in Germany examining PROMs use, with rheumatologists providing a mean rating for the subject of discussing results with patients of 38.1 on a scale of 0 (lowest importance) to 100 (highest importance). An important finding from our interviews was that patients advocated HCPs acknowledging ePROMs completion in some form.

Importantly, findings from the NPT-guided NoMAD survey offered insights into how implementation of similar models of care can be enhanced. Although the sample size was small, NoMAD responses indicated that while coherence and cognitive participation appeared well established, to enhance implementation, greater support is needed for ‘collective action’ or the operational work people do to enact practices around ePROMs. This includes the relational work in teams to build confidence and accountability with leadership and management support, alongside the integration of ePROMs within the broader context of protocols and procedures within a department. In addition, findings from NoMAD suggested that although participants seemed to have information to help them appraise and reflect on the use of ePROMs in their practice, systematisation and communal appraisal could be enhanced, e.g. with team meetings to discuss how ePROMs are being used service-wide.

Both patients and HCPs had a vision of how ePROMs could be applied to drive care improvements. These included using ePROMs to triage waiting lists (ensuring the right patients are seen at the right time, by the right person) and between-appointment monitoring. Such approaches have been used successfully in other studies [6, 23–25], and the high levels of ePROMs acceptability we observed indicate they could be applied to deliver this in routine NHS settings, providing innovative solutions to perennial issues such as long waiting lists. However, patients were clear that the use of ePROMs should not replace in-person appointments.

Our study has several strengths. First, its mixed methods design and involvement of both patients and HCPs ensured an in-depth exploration of ePROMs acceptability and implementation barriers. Second, using theoretical frameworks to guide acceptability and implementation evaluations added methodological rigour, ensuring the multifaceted dimensions of acceptability and implementation were considered and contextualised within established theoretical models. Third, by providing all patients with the opportunity to complete ePROMs (at home or supported in clinic) we optimised inclusivity and were able to explore ePROMs acceptability from the perspective of people unable to complete ePROMs on their own device.

Our study also has limitations. First, the sample size of HCPs and HAP non-completers was small. Second, our patient sample was predominantly of White ethnicity, and recruited from one Trust, potentially limiting the generalisability of the findings to more ethnically diverse populations. Attitudes towards digital health tools, health literacy and internet access may differ in areas with greater ethnic diversity or within other UK and international contexts. Third, HAP is a custom-built platform and the acceptability of ePROMs on other systems may not be the same, although our survey focused on the acceptability of answering online questions about health rather than the ePROMs delivery system.

In conclusion, using ePROMs in routine IA care is feasible and acceptable to patients and HCPs, supporting national initiatives for their widespread implementation to deliver efficient, effective patient care. As their acceptability is lower in patients who are older, less health literate and with lower digital skills/access, these factors require careful consideration in ePROMs implementation to avoid the unintentional worsening of health inequalities. Further research is needed to understand the best way to implement ePROMs in different NHS settings.

## Supplementary Material

rkag026_Supplementary_Data

## Data Availability

Keele University is a member of the UK Reproducibility Network and committed to the principles of the UK Concordat on Open Research Data. The School of Medicine had a long-standing commitment to sharing data from our studies to improve research reproducibility and to maximise benefits for patients, the wider public and the health and care system. We encourage collaboration with those who collected the data and recognise and credit their contributions. The School of Medicine makes data available to bona fide researchers upon reasonable request via open or restricted access through a strict controlled access procedure. The release of data may be subject to a data use agreement between the sponsor and the third party requesting the data. In the first instance, data requests and enquiries should be directed to: medicine.datasharing@keele.ac.uk.
