# Supplementary material for: Variation in the feasibility and acceptability of electronic patient-reported outcome measures in patients with inflammatory arthritis
Source: Rheumatol Adv Pract. 2026 Feb 17;10(2):rkag026. doi: 10.1093/rap/rkag026 (PMC13033184; doi:10.1093/rap/rkag026)
Supplement: rkag026_Supplementary_Data [file rkag026_supplementary_data.zip › Supplementary Data S1.pdf]

The Haywood Arthritis Portal Study

A questionnaire for patients completing the Haywood Arthritis Portal on their own digital device

INSTRUCTIONS FOR THIS QUESTIONNAIRE

- The aim of this questionnaire is to find out your views on answering online questions about your health (called "patient-reported outcome measures" or "PROMs" for short) and the Haywood Arthritis Portal.
- There are no right or wrong answers.
- For all questions, please check the relevant box (by placing a cross) to indicate your answer.
- If you are completing a paper questionnaire at home, **please check you have answered all questions** and return it in the pre-paid envelope enclosed. **No stamp is needed**.
- If you have any questions, or need help completing this questionnaire, please discuss this with the research nurse in clinic today, or if you are completing it at home, please telephone the Haywood Arthritis Portal research team on 07773 949356 or email them at hapstudy@mpft.nhs.uk.

Thank you very much for your help with this study.

Please note the date you are completing this questionnaire

☐☐	☐☐	☐☐☐☐
Day	Month	Year

SECTION 1: Your views on answering online questions about your health

The following questions are about how acceptable you found answering online questions about your health (called "PROMs").

Please check the <u>one box</u> for each statement that best indicates your opinion.

1. How acceptable was it to answer online questions about your health?

Completely unacceptable	Unacceptable	No opinion	Acceptable	Completely acceptable
☐	☐	☐	☐	☐

2. Did you like or dislike answering online questions about your health?

Strongly dislike	Dislike	No opinion	Like	Strongly like
☐	☐	☐	☐	☐

3. How much effort did it take to answer online questions about your health?

No effort at all	A little effort	No opinion	A lot of effort	Huge effort
☐	☐	☐	☐	☐

4. How fair (to all patients) is a system where patients are asked to answer online questions about their health (either at home or on a tablet in clinic)?

Very unfair	Unfair	No opinion	Fair	Very fair
☐	☐	☐	☐	☐

5. **My answers to the online questions about my health are likely to help my doctor or nurse treat me**

Strongly disagree	Disagree	No opinion	Agree	Strongly agree
☐	☐	☐	☐	☐

6. **It is clear to me how answering online questions about my health will help my doctor or nurse treat me**

Strongly disagree	Disagree	No opinion	Agree	Strongly agree
☐	☐	☐	☐	☐

7. **How confident did you feel about answering online questions about your health?**

Very unconfident	Unconfident	No opinion	Confident	Very confident
☐	☐	☐	☐	☐

8. **Completing online questions about my health interferes with my other priorities**

Strongly disagree	Disagree	No opinion	Agree	Strongly agree
☐	☐	☐	☐	☐

SECTION 2: If your online question answers were used at your appointment today

The following 3 statements are about whether you felt the doctor, nurse, or physiotherapist seeing you today used your answers to the online questions about your health.

Please check the one box for each statement that best indicates how much you agree or disagree with it.

	Strongly Disagree	Disagree	No opinion	Agree	Strongly agree
1. The doctor, nurse, or physiotherapist had looked at my online question answers.	☐	☐	☐	☐	☐
2. The doctor, nurse, or physiotherapist discussed my online question answers with me.	☐	☐	☐	☐	☐
3. The doctor, nurse, or physiotherapist used my online question answers to make decisions about my care.	☐	☐	☐	☐	☐

SECTION 3: If you feel that answering online questions about your health improved your care today

The following 2 statements are about whether you think that answering online questions about your health improved your care today.

Please check the one box for each statement that best indicates how much you agree or disagree with it.

	Strongly Disagree	Disagree	No opinion	Agree	Strongly agree
1. Answering the online questions helped me think about any problems I needed to discuss at my clinic appointment.	☐	☐	☐	☐	☐
2. Answering the online questions led to a better appointment with my doctor, nurse, or physiotherapist today	☐	☐	☐	☐	☐

SECTION 4: How easy you found the Haywood Arthritis Portal to use

The following 8 statements are about how easy you found the Haywood Arthritis Portal to use. This is the online system that you used to answer questions about your health.

Please check the <u>one box</u> for each statement that best indicates how much you agree or disagree with it.

	Strongly Disagree	Disagree	No opinion	Agree	Strongly agree
1. I am comfortable with my ability to use the Haywood Arthritis Portal	☐	☐	☐	☐	☐
2. Learning to use the Haywood Arthritis Portal is easy for me	☐	☐	☐	☐	☐
3. It is easy for me to become skilful at using the Haywood Arthritis Portal	☐	☐	☐	☐	☐
4. I find the Haywood Arthritis Portal easy to use	☐	☐	☐	☐	☐
5. I can always remember how to log on to and use the Haywood Arthritis Portal	☐	☐	☐	☐	☐
6. The Haywood Arthritis Portal gives error messages that clearly tell me how to fix problems	☐	☐	☐	☐	☐
7. Whenever I make a mistake using the Haywood Arthritis Portal, I recover easily and quickly	☐	☐	☐	☐	☐
8. The information (such as on-line help, on-screen messages, and other documentation) provided with the Haywood Arthritis Portal is clear	☐	☐	☐	☐	☐

SECTION 5: Your Use of the Internet

1. **Have you been able to access the internet (if you wanted to) in the last 3 months?**

Yes	No
☐	☐

2. **If you answered yes to question 1, please select all the ways that you have accessed the internet in the last 3 months (<u>you can select more than one option</u>)**

On my own computer	On my own smartphone	On my own tablet	On my own smartwatch	On a friend's or relative's computer, smartphone, or tablet	On a public computer, tablet, or smartphone (e.g., in a library)
☐	☐	☐	☐	☐	☐

3. **How often have you used the internet in the last 3 months (please select the <u>one answer</u> that best describes your internet use)?**

Every day or almost every day	Every week but not every day	Less than once a week	I have not used it in the last 3 months
☐	☐	☐	☐

SECTION 6: Your Use of the Internet to Help with Your Health Problems

We would like to ask you for your opinion and about your experience using the internet for health information. For each statement, please tell us which response best reflects your opinion and experience right now.

1. How **useful** do you feel the Internet is in helping you in making decisions about your health?

Not useful at all	Not useful	Unsure	Useful	Very useful
☐	☐	☐	☐	☐

2. How **important** is it for you to be able to access health resources on the Internet?

Not important at all	Not important	Unsure	Important	Very important
☐	☐	☐	☐	☐

	Strongly Disagree	Disagree	No opinion	Agree	Strongly agree
3. I know **what** health resources are available on the internet	☐	☐	☐	☐	☐
4. I know **where** to find helpful health resources on the internet	☐	☐	☐	☐	☐
5. I know **how** to find helpful health resources on the internet	☐	☐	☐	☐	☐
6. I know **how to use** the internet to answer my questions about health	☐	☐	☐	☐	☐
7. I know how to use **the health information** I find on the internet to help me	☐	☐	☐	☐	☐
8. I have the skills I need to **evaluate** the health resources I find on the internet	☐	☐	☐	☐	☐
9. I can tell **high quality** health resources from **low quality** health resources on the internet	☐	☐	☐	☐	☐
10. I feel **confident** in using information from the internet to make health decisions	☐	☐	☐	☐	☐

SECTION 7: Your Digital Skills

The next 7 questions assess your ability to use digital "devices" that can connect to the internet (such as a computer, smartphone, tablet, or smartwatch).

Please read each statement and answer "yes" if you can do it or "no" if you cannot do it.

	Yes	No
1. I can use the available controls on a device (e.g., a mouse, keyboard, touchscreen etc.)	☐	☐
2. I can open an internet browser to find and use websites	☐	☐
3. I can turn on a device and log in to any accounts/profiles I have	☐	☐
4. I can find and open different applications/programmes on a device	☐	☐
5. I can update and change my password when prompted to do so	☐	☐
6. I can connect a device to a Wi-Fi network	☐	☐
7. I can use the different menu settings on a device to make it easier to use (e.g., change the font size to make it easier to read)	☐	☐

SECTION 8: About You

1. **What is your ethnic group? (please check the one box that best describes your ethnic group or background)**

A. White

☐ Welsh/English/Scottish/Northern Irish/British
☐ Irish
☐ Gypsy or Irish Traveller
☐ Any other White background, please write on the line below what this is

____

B. Mixed/Multiple ethnic groups

☐ White and Black Caribbean

☐ White and Black African

☐ White and Asian

☐ Any other Mixed/Multiple ethnic background, please write on the line below what this is

____

C. Asian/Asian British

☐ Indian

☐ Pakistani

☐ Bangladeshi

☐ Chinese

☐ Any other Asian background, please write on the line below what this is

____

D. Black/African/Caribbean/Black British

☐ African

☐ Caribbean

☐ Any other Black/African/Caribbean, please write on the line below what this is

____

E. Other Ethnic Group

☐ Arab

☐ Any other ethnic group, please write on the line below what this is

____

2. What is your postcode?

3. Which of the following best describes your employment status? (Please check one box only)

Employed/self-employed	☐	Retired due to ill health	☐
Not working due to ill health	☐	Unemployed or seeking work	☐
Student	☐	Volunteer work	☐
Retired	☐	Other (please write what this is on the line)	☐ ____________ ____________
Looking after children/home	☐		

4. If you are working, what is your job title, or if you are not working or are retired, what was your last job title? (Please write this on the below line)

____

5. How often do you need to have someone help you when you read instructions on pamphlets, or other written material from your doctor or pharmacy?

Never	Rarely	Sometimes	Often	Always

SECTION 9: Any Other Information You Wish to Tell Us About Answering Online Questions About Health

Please write on the line below if there is anything else you would like to tell us about answering online questions about your health or the Haywood Arthritis Portal that has not been covered by the questions so far.

____

____

____

____

THANK YOU FOR COMPLETING THIS QUESTIONNAIRE

Please check you have answered all questions and return it in the pre-paid envelope enclosed. No stamp is needed.

Study ID:
