# Supplementary material for: Variation in the feasibility and acceptability of electronic patient-reported outcome measures in patients with inflammatory arthritis
Source: Rheumatol Adv Pract. 2026 Feb 17;10(2):rkag026. doi: 10.1093/rap/rkag026 (PMC13033184; doi:10.1093/rap/rkag026)
Supplement: rkag026_Supplementary_Data [file rkag026_supplementary_data.zip › Supplementary Data S3.pdf]

The Haywood Arthritis Portal Study

A questionnaire for patients preferring not to complete the Haywood Arthritis Portal

INSTRUCTIONS FOR THIS QUESTIONNAIRE

- The aim of this questionnaire is to find out your views on answering online questions about your health (called "patient-reported outcome measures" or "PROMs" for short) and the Haywood Arthritis Portal.

- There are no right or wrong answers.

- For all questions, please check the relevant box (by placing a cross) to indicate your answer.

- If you are completing a paper questionnaire at home, **please check you have answered all questions** and return it in the pre-paid envelope enclosed. **No stamp is needed**.

- If you have any questions, or need help completing this questionnaire, please discuss this with the research nurse in clinic today, or if you are completing it at home, please telephone the Haywood Arthritis Portal research team on 07773 949356 or email them at hapstudy@mpft.nhs.uk.

Thank you very much for your help with this study.

Please note the date you are completing this questionnaire

Day		Month		Year			

SECTION 1: Why you didn't complete the Haywood Arthritis Portal

1. **Did you receive a text message or a letter about completing the Haywood Arthritis Portal before your appointment today?**

Yes	No
☐	☐

2. **We would like to know the reason why you preferred not to complete the Haywood Arthritis Portal on tablets in clinic today. If you are happy to tell us, please write the reason for this on the lines below.**

____

____

____

SECTION 2: Your Use of the Internet

1. **Have you been able to access the internet (if you wanted to) in the last 3 months?**

Yes	No
☐	☐

2. **If you answered yes to question 1, please select all the ways that you have accessed the internet in the last 3 months (<u>you can select more than one option</u>)**

On my own computer	On my own smartphone	On my own tablet	On my own smartwatch	On a friend's or relative's computer, smartphone, or tablet	On a public computer, tablet, or smartphone (e.g., in a library)
☐	☐	☐	☐	☐	☐

3. **How often have you used the internet in the last 3 months (please select the <u>one answer</u> that best describes your internet use)?**

Every day or almost every day	Every week but not every day	Less than once a week	I have not used it in the last 3 months
☐	☐	☐	☐

SECTION 3: Your Use of the Internet to Help with Your Health Problems

We would like to ask you for your opinion and about your experience using the internet for health information. For each statement, please tell us which response best reflects your opinion and experience <u>right now</u>.

1. How **useful** do you feel the Internet is in helping you in making decisions about your health?

Not useful at all	Not useful	Unsure	Useful	Very useful
☐	☐	☐	☐	☐

2. How **important** is it for you to be able to access health resources on the Internet?

Not important at all	Not important	Unsure	Important	Very important
☐	☐	☐	☐	☐

	Strongly Disagree	Disagree	No opinion	Agree	Strongly agree
3. I know **what** health resources are available on the internet	☐	☐	☐	☐	☐
4. I know **where** to find helpful health resources on the internet	☐	☐	☐	☐	☐
5. I know **how** to find helpful health resources on the internet	☐	☐	☐	☐	☐
6. I know **how to use** the internet to answer my questions about health	☐	☐	☐	☐	☐
7. I know how to use **the health information I** find on the internet to help me	☐	☐	☐	☐	☐
8. I have the skills I need to **evaluate** the health resources I find on the internet	☐	☐	☐	☐	☐
9. I can tell **high quality** health resources from **low quality** health resources on the internet	☐	☐	☐	☐	☐
10. I feel **confident** in using information from the internet to make health decisions	☐	☐	☐	☐	☐

SECTION 4: Your Digital Skills

The next 7 questions assess your ability to use digital “devices” that can connect to the internet (such as a computer, smartphone, tablet, or smartwatch).

Please read each statement and answer “yes” if you can do it or “no” if you cannot do it.

	Yes	No
1. I can use the available controls on a device (e.g., a mouse, keyboard, touchscreen etc.)	☐	☐
2. I can open an internet browser to find and use websites	☐	☐
3. I can turn on a device and log in to any accounts/profiles I have	☐	☐
4. I can find and open different applications/programmes on a device	☐	☐
5. I can update and change my password when prompted to do so	☐	☐
6. I can connect a device to a Wi-Fi network	☐	☐
7. I can use the different menu settings on a device to make it easier to use (e.g., change the font size to make it easier to read)	☐	☐

SECTION 5: About You

1. What is your ethnic group? (please check the one box that best describes your ethnic group or background)

A. White

☐ Welsh/English/Scottish/Northern Irish/British

☐ Irish

☐ Gypsy or Irish Traveller

☐ Any other White background, please write on the line below what this is

____

B. Mixed/Multiple ethnic groups

- ☐ White and Black Caribbean
- ☐ White and Black African
- ☐ White and Asian
- ☐ Any other Mixed/Multiple ethnic background, please write on the line below what this is

____

C. Asian/Asian British

- ☐ Indian
- ☐ Pakistani
- ☐ Bangladeshi
- ☐ Chinese
- ☐ Any other Asian background, please write on the line below what this is

____

D. Black/African/Caribbean/Black British

- ☐ African
- ☐ Caribbean
- ☐ Any other Black/African/Caribbean, please write on the line below what this is

____

E. Other Ethnic Group

- ☐ Arab
- ☐ Any other ethnic group, please write on the line below what this is

____

2. What is your postcode?

3. Which of the following best describes your employment status? (Please check one box only)

Employed/self-employed	☐	Retired due to ill health	☐
Not working due to ill health	☐	Unemployed or seeking work	☐
Student	☐	Volunteer work	☐
Retired	☐	Other (please write what this is on the line)	☐ ________ ________
Looking after children/home	☐		

4. If you are working, what is your job title, or if you are not working or are retired, what was your last job title? (Please write this on the below line)

____

5. How often do you need to have someone help you when you read instructions on pamphlets, or other written material from your doctor or pharmacy?

Never	Rarely	Sometimes	Often	Always

THANK YOU FOR COMPLETING THIS QUESTIONNAIRE

Please check you have answered all questions and return it in the pre-paid envelope enclosed. No stamp is needed.

Study ID:
