# Supplementary material for: Variation in the feasibility and acceptability of electronic patient-reported outcome measures in patients with inflammatory arthritis
Source: Rheumatol Adv Pract. 2026 Feb 17;10(2):rkag026. doi: 10.1093/rap/rkag026 (PMC13033184; doi:10.1093/rap/rkag026)
Supplement: rkag026_Supplementary_Data [file rkag026_supplementary_data.zip › Supplementary Data S4.pdf]

HAP: Healthcare Professional Questionnaire Version 2.0

The Haywood Arthritis Portal Study.

Healthcare Professional Questionnaire Version 2.0

Questionnaire Instructions

- This questionnaire seeks to understand your views on the use of electronic patient-reported outcome measures (ePROMs) in the care of patients with inflammatory arthritis, alongside the Haywood Arthritis and Rheumatology Portals.

- For all questions, please select the relevant option to indicate your answer.

- There are no right or wrong answers.

- This questionnaire has several pages.

- Questions with a red asterix * need to be answered.

- At the top of each page is a "progress bar". This shows how much of the questionnaire you have completed (from 0% to 100%).

- Once you have answered all the questions on a page, please click the "Next" button at the bottom to continue.

- If you would like to go back to a previous page (for example to change an answer) please click on the "Previous" button at the bottom.

- Once you have completed all questions and you are happy with your answers, please click the "Submit" button at the end of the questionnaire.

- The questionnaire takes around 10 minutes to complete.

- All questions need to be answered in one go without leaving the web page.

- If you have any questions, or need help completing this online questionnaire, please contact the HAP Project team on 07773949356 or email at hapstudy@mpft.nhs.uk.

Thank you very much for your help with this study.

There are 49 questions in this survey.

Type of Inflammatory Arthritis consultations you have undertaken in which ePROMs were available

Todays Date: **{date('d-m-Y')}**

1. Please select which type(s) of inflammatory arthritis consultations you have undertaken in which ePROMs data were available. Your answer will determine the questions you will be asked. Please select **all** that apply. *

Check all that apply

Please choose **all** that apply:

- [ ] Rheumatoid Arthritis
- [ ] Axial Spondyloarthritis
- [ ] Psoriatic Arthritis or other peripheral Spondyloarthritis
- [ ] Other type(s) of Inflammatory Arthritis

Section 1: Your views on the acceptability of ePROMs

The following questions are about how acceptable you have found having ePROMs data available during consultations for patients with inflammatory arthritis.

For each question, please select **the one response** that best indicates your answer:

1. How acceptable was it having ePROMs data available? *

Please choose **only one** of the following:

- ○ Completely unacceptable
- ○ Unacceptable
- ○ No opinion
- ○ Acceptable
- ○ Completely acceptable

2. Did you like or dislike having ePROMs data available? *

Please choose **only one** of the following:

- ◯ Strongly dislike
- ◯ Dislike
- ◯ No opinion
- ◯ Like
- ◯ Strongly like

3. How much effort did it take you to use ePROMs data? *

Please choose **only one** of the following:

- ◯ No effort at all
- ◯ A little effort
- ◯ No opinion
- ◯ A lot of effort
- ◯ Huge effort

4. There are moral or ethical consequences to using ePROMs data *

Please choose **only one** of the following:

- ◯ Strongly disagree
- ◯ Disagree
- ◯ No opinion
- ◯ Agree
- ◯ Strongly agree

5. ePROMs data are likely to improve the care patients with inflammatory arthritis receive *

Please choose **only one** of the following:

- ◯ Strongly disagree
- ◯ Disagree
- ◯ No opinion
- ◯ Agree
- ◯ Strongly agree

6. It makes sense to me how ePROMs data will result in improvements in the care patients with inflammatory arthritis receive *

Please choose **only one** of the following:

- ◯ Strongly disagree
- ◯ Disagree
- ◯ No opinion
- ◯ Agree
- ◯ Strongly agree

7. How confident did you feel about using ePROMs data? *

Please choose **only one** of the following:

- ◯ Very unconfident
- ◯ Unconfident
- ◯ No opinion
- ◯ Confident
- ◯ Very confident

8. Using ePROMs data interferes with my other priorities *

Please choose **only one** of the following:

- ◯ Strongly disagree
- ◯ Disagree
- ◯ No opinion
- ◯ Agree
- ◯ Strongly agree

Section 2: Your views on the ePROMs captured by the Haywood Arthritis Portal

For each question, please select **the one response** that best indicates your answer. (Please note some questions may be missing depending on the types of inflammatory arthritis consultations you reported undertaking at the start of the survey)

1. Do you feel the Haywood Arthritis Portal captures the right amount of ePROMs data from patients? *

Please choose **only one** of the following:

- ◯ No - too little
- ◯ No - too much
- ◯ Yes - the right amount

2. In patients with rheumatoid arthritis, I find the following ePROMs useful *

Please choose the appropriate response for each item:

	**Strongly disagree**	**Disagree**	**No opinion**	**Agree**	**Strongly agree**
**A. 28 patient swollen joint counts**	◯	◯	◯	◯	◯
**B. 28 patient tender joint counts**	◯	◯	◯	◯	◯

3. In patients with peripheral arthritis (e.g. rheumatoid arthritis, peripheral spondyloarthritis), I find the following ePROMs useful *

Please choose the appropriate response for each item:

	Strongly disagree	Disagree	No opinion	Agree	Strongly agree
**A. Patient global assessment**	◯	◯	◯	◯	◯
**B. Pain intensity scores**	◯	◯	◯	◯	◯
**C. Analgesic use**	◯	◯	◯	◯	◯
**D. HAQ**	◯	◯	◯	◯	◯
**E. RAPID3**	◯	◯	◯	◯	◯
**F. MSK-HQ**	◯	◯	◯	◯	◯

4. In patients with axial spondyloarthritis, I find the following ePROMs useful *

Please choose the appropriate response for each item:

	Strongly disagree	Disagree	No opinion	Agree	Strongly agree
**A. Patient global assessment**	○	○	○	○	○
**B. Pain intensity scores**	○	○	○	○	○
**C. Analgesic use**	○	○	○	○	○
**D. BASDAI**	○	○	○	○	○
**E. BASFI**	○	○	○	○	○
**F. MSK-HQ**	○	○	○	○	○

5. Patient completed joint counts in rheumatoid arthritis are accurate *

Please choose **only one** of the following:

- ○ Strongly disagree
- ○ Disagree
- ○ No opinion
- ○ Agree
- ○ Strongly agree

6. The RAPID3 score is an accurate measure of disease activity in patients with rheumatoid arthritis *

Please choose **only one** of the following:

- ◯ Strongly disagree
- ◯ Disagree
- ◯ No opinion
- ◯ Agree
- ◯ Strongly agree

Section 3: How you use ePROMs data in face-to-face consultations

The next four questions are about how you use ePROMs data (when available) during **face-to-face consultations** for patients with inflammatory arthritis**.**

For each question, please select the **one response** that best indicates your answer**.**

1. I look at ePROMs data that patients have entered *

Please choose **only one** of the following:

- ◯ Never
- ◯ Rarely
- ◯ Sometimes
- ◯ Often
- ◯ Always

2. I discuss ePROMs data that patients have entered with them *

Please choose **only one** of the following:

- Never
- Rarely
- Sometimes
- Often
- Always

3. I use patients' ePROMs data to help make decisions on their care *

Please choose **only one** of the following:

- Strongly disagree
- Disagree
- No opinion
- Agree
- Strongly agree

4. ePROMs data have helped me identify issues I would otherwise not have known about (such as patients having depression and/or anxiety) *

Please choose **only one** of the following:

- ◯ Strongly disagree
- ◯ Disagree
- ◯ No opinion
- ◯ Agree
- ◯ Strongly agree

Section 4: How you use ePROMs data in remote consultations

1. Have you undertaken remote consultations for patients with inflammatory arthritis where ePROMs data were available? *

Please choose **only one** of the following:

- ◯ Yes
- ◯ No

The next six questions are about how you use ePROMs data (when available) during **remote consultations** for patients with inflammatory arthritis.

For each question, please select the **one response** that best indicates your answer.

2. I look at ePROMs data that patients have entered *

Please choose **only one** of the following:

- Never
- Rarely
- Sometimes
- Often
- Always

3. I discuss ePROMs data that patients have entered with them *

Please choose **only one** of the following:

- Never
- Rarely
- Sometimes
- Often
- Always

4. I use patients' ePROMs data to help me make decisions on their care *

Please choose **only one** of the following:

- Strongly disagree
- Disagree
- No opinion
- Agree
- Strongly agree

5. Having ePROMs data available makes it less likely I will organise to see a patient face-to-face *

Please choose **only one** of the following:

- ◯ Strongly disagree
- ◯ Disagree
- ◯ No opinion
- ◯ Agree
- ◯ Strongly agree

6. I feel comfortable making decisions about immunosuppression based on ePROMs measured disease activity (e.g., patient joint counts and RAPID3 scores) in patients with rheumatoid arthritis *

Please choose **only one** of the following:

- ◯ Strongly Disagree
- ◯ Disagree
- ◯ No opinion
- ◯ Agree
- ◯ Strongly agree
- ◯ Not relevant - I have not conducted RA remote consultations

7. I feel comfortable making decisions about immunosupression based on ePROMs measured disease activity (e.g., BASDAI) in patients with an axial spondyloarthritis *

Please choose **only one** of the following:

- ◯ Strongly disagree
- ◯ Disagree
- ◯ No opinion
- ◯ Agree
- ◯ Strongly agree
- ◯ Not relevant - I have not conducted axial SpA remote consultations

Section 5: Understanding how ePROMs data can be integrated into routine care

The following questions are designed to help get a better understanding of how to apply and integrate new technologies and complex interventions in health care.

They ask about the implementation of ePROMs data (captured using the Haywood Arthritis Portal) during consultations for patients with inflammatory arthritis.

Please answer all questions from the perspective of your role in relation to using ePROMs data.

The questions are in 2 parts:

- Part A - includes three general questions about ePROMs in consultations for patients with inflammatory arthritis.
- Part B - contains a set of of more detailed questions about using ePROMs in consultations for patients with inflammatory arthritis.

Please take the time to decide which answer **best suits your experience for each statement and choose the appropriate response.**

Part A: general questions about using ePROMs - please select a response on a scale of 0 - 10 for each question.

1. When you use ePROMs, **how familiar** does it feel? *

Please choose the appropriate response for each item:

	0	1	2	3	4	5	6	7	8	9	10
	○	○	○	○	○	○	○	○	○	○	○

Where 0 = Still feels very new, and 10 = Feels completely familiar

2. Do you feel ePROMs **are currently** a normal part of your work? *

Please choose the appropriate response for each item:

	0	1	2	3	4	5	6	7	8	9	10
	○	○	○	○	○	○	○	○	○	○	○

Where 0 = Not at all, 5 = Somewhat, and 10 = Completely

3. Do you feel ePROMs **will become** a normal part of your work? *

Please choose the appropriate response for each item:

	0	1	2	3	4	5	6	7	8	9	10
	○	○	○	○	○	○	○	○	○	○	○

Where 0 = Not at all, 5 = Somewhat, and 10 = Completely

Section 5B: Understanding how ePROMs data can be integrated into routine care

Part B: Detailed questions about using ePROMs.

1. For each statement, please select an answer that best indicates how much you agree or disagree with it. *

Please choose the appropriate response for each item:

	Strongly disagree	Disagree	Neither agree nor disagree	Agree	Strongly agree
**A. I can see how having ePROMs differs from usual ways of working**	○	○	○	○	○
**B. Staff in this organisation have a shared understanding of the purpose of ePROMs**	○	○	○	○	○
**C. I understand how having ePROMs affects the nature of my own work**	○	○	○	○	○
**D. I can see the potential value of ePROMs for my work**	○	○	○	○	○
**E. There are key people who drive the use of ePROMs forward at our NHS trust and get others involved**	○	○	○	○	○
**F. I believe that participating in using ePROMs is a legitimate part of my role**	○	○	○	○	○

	Strongly disagree	Disagree	Neither agree nor disagree	Agree	Strongly agree
**G. I'm open to working with colleagues in new ways to use ePROMs**	◯	◯	◯	◯	◯
**H. I will continue to support the use of ePROMs at our NHS trust**	◯	◯	◯	◯	◯
**I. I can easily integrate using ePROMs into my existing work**	◯	◯	◯	◯	◯
**J. Using ePROMs disrupts working relationships**	◯	◯	◯	◯	◯
**K. I have confidence in other people's ability to use ePROMs**	◯	◯	◯	◯	◯
**L. Work is assigned to those with skills appropriate to use ePROMs**	◯	◯	◯	◯	◯
**M. Sufficient training is provided to enable staff to use ePROMs**	◯	◯	◯	◯	◯
**N. Sufficient resources are available to support ePROMs**	◯	◯	◯	◯	◯
**O. Management adequately supports ePROMs**	◯	◯	◯	◯	◯

	Strongly disagree	Disagree	Neither agree nor disagree	Agree	Strongly agree
**P. I am aware of reports about the effects of ePROMs**	○	○	○	○	○
**Q. The staff agree that having ePROMs is worthwhile**	○	○	○	○	○
**R. I value the effects that ePROMs data has had on my work**	○	○	○	○	○
**S. Feedback about the Haywood Arthritis Portal can be used to improve it in the future**	○	○	○	○	○
**T. I can modify how I work with ePROMs data**	○	○	○	○	○

Section 6: How easy you found the Haywood Rheumatology Portal to use

The Haywood Rheumatology Portal is the electronic health record system that allows you to view ePROMs data, enter other outcome data (e.g., DAS28 scores), complete consultation outcome forms, and request rheumatology ultrasound scan tests.

1. The following 6 statements are about how easy you found the Haywood Rheumatology Portal to use.

Please click **one box** for each statement that best indicates how much you agree or disagree with it. *

Please choose the appropriate response for each item:

	Strongly disagree	Disagree	No opinion	Agree	Strongly agree
**A. I am comfortable with my ability to use the Haywood Rheumatology Portal**	◯	◯	◯	◯	◯
**B. Learning to use the Haywood Rheumatology Portal is easy for me**	◯	◯	◯	◯	◯
**C. It is easy for me to become skilful at using the Haywood Rheumatology Portal**	◯	◯	◯	◯	◯
**D. I find the Haywood Rheumatology Portal easy to use**	◯	◯	◯	◯	◯
**E. Whenever I make a mistake using the Haywood Rheumatology Portal, I recover easily and quickly**	◯	◯	◯	◯	◯

	Strongly disagree	Disagree	No opinion	Agree	Strongly agree
**F. The information (such as on-line help, on-screen messages, and other documentation) provided with the Haywood Rheumatology Portal is clear**	◯	◯	◯	◯	◯

Section 7: About you

1. What is your current age? *

Please choose **only one** of the following:

- ◯ <30 years
- ◯ 30 - <40 years
- ◯ 40 - <50 years
- ◯ 50 - <60 years
- ◯ ≥60 years
- ◯ Prefer not to say

2. What was your sex at birth? *

Please choose **only one** of the following:

- ◯ Male
- ◯ Female
- ◯ Prefer not to say

3. What is your ethnic group? (please select the one option that best describes your ethnic group or background - you will then be able to choose from more detailed ethnic group options) *

Please choose **only one** of the following:

- ◯ White
- ◯ Mixed / Multiple ethnic groups
- ◯ Asian/ Asian British
- ◯ Black / African / Caribbean / Black British
- ◯ Other ethnic group
- ◯ Prefer not to say

A. White: Please now select one option to best describe your ethnic group or background. *

Please choose **only one** of the following:

- ◯ English / Welsh / Scottish / Northern Irish / British
- ◯ Irish
- ◯ Gypsy or Irish Traveller
- ◯ Other ________

B. Mixed / Multiple ethnic groups: Please now select one option to best describe your ethnic group or background. *

Please choose **only one** of the following:

- ◯ White and Black Caribbean
- ◯ White and Black African
- ◯ White and Asian
- ◯ Other ____________

C. Asian / Asian British: Please now select one option to best describe your ethnic group or background. *

Please choose **only one** of the following:

- ◯ Indian
- ◯ Pakistani
- ◯ Bangladeshi
- ◯ Chinese
- ◯ Other ____________

D. Black / African / Caribbean / Black British: Please now select one option to best describe your ethnic group or background.
*

Please choose **only one** of the following:

- ◯ African
- ◯ Caribbean
- ◯ Other [ ]

E. Other ethnic group: Please now select one option to best describe your ethnic group or background.
*

Please choose **only one** of the following:

- ◯ Arab
- ◯ Other [ ]

4. What is your medical occupation? *

Please choose **only one** of the following:

- ◯ Consultant
- ◯ Specialist registrar
- ◯ Staff grade
- ◯ Internal medical trainee
- ◯ Foundation year doctor
- ◯ Extended scope physiotherapist
- ◯ Clinical nurse specialist
- ◯ Prefer not to say

5. How long have you been working as a healthcare professional? *

Please choose **only one** of the following:

- ◯ <5 years
- ◯ 5 to 10 years
- ◯ 11 to 15 years
- ◯ 16 to 20 years
- ◯ >20 years

6. How many years have you worked in the Midlands Partnership NHS Foundation Trust Rheumatology Department? *

Please choose **only one** of the following:

- ◯ <1 year
- ◯ 1 - 2 years
- ◯ 3 - 5 years
- ◯ 6 - 10 years
- ◯ 11 - 15 years
- ◯ >15 years

Section 8: Any other information not previously covered

1. Is there anything else you would like to tell us about ePROMs?

Please write your answer here:

2. Is there anything else you would like to tell us about the Haywood Rheumatology Portal?

Please write your answer here:

3. Is there anything else you would like to tell us about the Haywood Arthritis Portal?

Please write your answer here:

Your answers have been successfully submitted.

If you have any questions or need help completing this online questionnaire, please contact the HAP team on 07773949356 or email at hapstudy@mpft.nhs.uk.

Thank you very much for your help with this study.

16.05.2024 – 15:31

Submit your survey.

Thank you for completing this survey.
