# Supplementary material for: Variation in the feasibility and acceptability of electronic patient-reported outcome measures in patients with inflammatory arthritis
Source: Rheumatol Adv Pract. 2026 Feb 17;10(2):rkag026. doi: 10.1093/rap/rkag026 (PMC13033184; doi:10.1093/rap/rkag026)
Supplement: rkag026_Supplementary_Data [file rkag026_supplementary_data.zip › Supplementary Data S5.pdf]

The Haywood Arthritis Portal Study

Semi-Structured Interview Schedule for Patient Participants

Portal Completers

Housekeeping

- Welcome and introductions
- Informed consent and consent to record interview
- Check understanding of how to use Microsoft Teams for videocall interviews
- Take breaks as and when needed
- If connection of telephone or videocall fails, interviewer will contact them again
- Collect background information (e.g. age, where they live, current or previous occupation)

Introduction

- You have been invited here because you should have recently been asked to answer some online questions about your health and arthritis using something called the 'Haywood Arthritis Portal' before an appointment for your inflammatory arthritis, is that right?
- At your arthritis appointment, you should have been asked to complete a research survey. **Today, we are interested in learning about your experiences of using the Haywood Arthritis Portal (not to be mixed up with the survey).**
- The reason we are running this study is because we want to know what patients with inflammatory arthritis think about using the Haywood Arthritis Portal and about answering online questions about their health in general. We would also like to know your thoughts on whether the Haywood Arthritis Portal should be used in the NHS as part of usual patient care.
- Please speak freely as both positive and negative answers are valued. There are no right and wrong answers. If you do not feel comfortable answering any questions, feel free to skip them, or you can choose to stop the interview altogether if you wish.
- Double check if participant is happy to continue with interview.

Section 1: Views on answering online health questions using the Haywood Arthritis Portal

- Did you complete a Haywood Arthritis Portal entry before your clinic appointment?
    - How many times did you complete an entry?
    - What type of healthcare professional(s) did you see for this/these clinic appointment(s)?
    - Did you use the Haywood Arthritis Portal on your own electronic device before your appointment or on a tablet in clinic?
        - *[If multiple entries have been completed]:* Did you use this method of completing the Haywood Arthritis Portal entry each time?
    - *[In-clinic completers only]:* Why did you not complete it on your own device?

- What did you think the Haywood Arthritis Portal was originally for?
    - Why did you think this?
    - Did your experience meet your expectations? *Tell us more about your experience…*
    - What could have made the experience better?

- Did you question whether or not to complete the Haywood Arthritis Portal before you did it?
    - What were your thoughts on this?
    - Why did you decide to complete it?

- Did you have any concerns about entering online information into the Haywood Arthritis Portal?
    - Why did you think this?

- How did you feel about answering online questions about your health before your appointment?
    - Why did you feel that way?

- How confident did you feel when using the Haywood Arthritis Portal?
    - Can you tell me why you felt that way?
    - What would've made you feel more confident?

- How were you notified about the Haywood Arthritis Portal?
    - What are your thoughts on this?
    - How hard was it to register for the Haywood Arthritis Portal?
    - What would have made it easier? (do you have any suggestions for improvement?)

- *[In-clinic completers only]:* How did you feel about being asked to complete the Haywood Arthritis Portal in the clinic whilst waiting for your appointment?
    - Can you tell me why you feel this way?

- What are your thoughts on the content of the portal?
    - Why do you think this?
    - Is there anything that could be done to improve the content of the portal?

- In general, how do you feel answering online health questions impacts your arthritis care?
    - Can you tell me why you feel this way?
    - What could be done to make this better?

- How do you feel the answers you provided in the Haywood Arthritis Portal were used by the healthcare professional treating you?
    - *[If multiple entries have been completed]:* How were your Haywood Arthritis Portal entries used differently by each healthcare professional?

- Do you think using the Haywood Arthritis Portal changed the care you received?
    - Why do you think this?
    - Can you tell me how this made you feel?

- What drawbacks does using the Haywood Arthritis Portal have?
    - Why do you think this?
    - Do you have any suggestions for improvements?

- In general, do you think that asking people to complete online questions about their health before their appointment is fair for all people with arthritis?
    - Can you tell me why you feel that way?
    - What could be done to make this fairer?

Section 2: Use of the Haywood Arthritis Portal in usual care

- How would you feel if the Haywood Arthritis Portal became part of your usual care?
    - Why do you feel this way? *[if negative feelings, what would be preferred instead?]*
    - If the Haywood Arthritis Portal became part of your usual care, is there anything that could make this better for you?

- What are your thoughts about completing the Haywood Arthritis Portal before every appointment?
    - Why do you feel this way? *[if negative, how often would be preferred?]*

- How would you feel about completing the Haywood Arthritis Portal between appointments?
    - Why do you feel this way?
    - What would make this better?

- What different ways do you think the Haywood Arthritis Portal *could* be used to deliver care to people with inflammatory arthritis? (E.g. to triage people on waiting lists)
    - Could you describe any potential positives or negatives to this?

Closing statement

- Do you have any questions or final comments you would like to mention?
- Thank you for participating. If you do have any further questions, please do let us know using the contact details on the participant information form.
