# Supplementary material for: Variation in the feasibility and acceptability of electronic patient-reported outcome measures in patients with inflammatory arthritis
Source: Rheumatol Adv Pract. 2026 Feb 17;10(2):rkag026. doi: 10.1093/rap/rkag026 (PMC13033184; doi:10.1093/rap/rkag026)
Supplement: rkag026_Supplementary_Data [file rkag026_supplementary_data.zip › Supplementary Data S6.pdf]

Keele UNIVERSITY

The Haywood Arthritis Portal Study

Semi-Structured Interview Schedule for Patient Participants

Portal Non-Completers

Housekeeping

- Welcome and introductions
- Informed consent and consent to record interview
- Check understanding of how to use Microsoft Teams for videocall interviews
- Take breaks as and when needed
- If connection of telephone or videocall fails, interviewer will contact them again
- Collect background information (e.g. age, where they live, current or previous occupation)

Introduction

- You have been invited here because you should have recently been asked to answer some online questions about your health and arthritis using something called the 'Haywood Arthritis Portal' before an appointment for your inflammatory arthritis, is that right?
    - And for your own reasons, you did not complete this, is that right?
- At your arthritis appointment, you should have been asked to complete a research survey. **Today, we are interested in learning about your views of the Haywood Arthritis Portal (not to be mixed up with the survey).**
- The reason we are running this study is because we want to know what patients with inflammatory arthritis think about using the Haywood Arthritis Portal and about answering online questions about their health in general. We would also like to know your thoughts on whether the Haywood Arthritis Portal should be used in the NHS as part of usual patient care.
- Please speak freely as both positive and negative answers are valued. There are no right and wrong answers. If you do not feel comfortable answering any questions, feel free to skip them, or you can choose to stop the interview altogether if you wish.
- Double check if participant is happy to continue with interview.

Section 1: Views on answering online health questions using the Haywood Arthritis Portal

- What, if any, experience do you have in using the Haywood Arthritis Portal?

- What did you think the Haywood Arthritis Portal was for? Why?

- Why did you choose not to complete a portal entry?
    - What could have been done to change this?

- Did you have any concerns about entering online information into the Haywood Arthritis Portal?
    - Why did you think this?

- How do you feel about answering online questions about your health before your rheumatology appointment?

    - Why do you feel this way?

- How confident do you feel about answering online questions about your health?
    - Can you tell me why you feel this way?
    - What would make you feel more confident?

- How were you notified about the Haywood Arthritis Portal?
    - What are your thoughts on this?
    - Did you attempt to register for the Haywood Arthritis Portal?
        - [*If yes]:* did you find it hard? what would have made it easier? *(any suggestions for improvement?)*

Information on the Haywood Arthritis Portal to be provided by interviewer:
Since you did not complete the Haywood Arthritis Portal, I will give you a brief description of what it is to help you answer some of the upcoming questions. The Haywood Arthritis Portal involves a set of online questions which allows people with inflammatory arthritis to report how their arthritis has been before their appointment. The intended purpose of this is to help the healthcare professionals treating them better understand how their arthritis is affecting them, also allowing for extra time in their appointments to discuss other things.

- How do you think using the Haywood Arthritis Portal could change the care you receive?
    - Why do you think this?
    - How does this make you feel?

- In general, how do you feel answering online health questions could impact your arthritis care?
    - Can you tell me why you feel this way?
    - What could be done to make this better?

- In general, do you think that asking people to complete online questions about their health before their appointment is fair for all people with arthritis?
    - Can you tell me why you feel that way?
    - What could be done to make this fairer?

Section 2: Use of the Haywood Arthritis Portal in usual care

- How would you feel if the Haywood Arthritis Portal became part of your usual care?
    - Why do you feel this way? *[if negative feelings, what would be preferred instead?]*
    - If the Haywood Arthritis Portal became part of your usual care, is there anything that could make this better for you?

- What are your thoughts about completing the Haywood Arthritis Portal before every appointment?
  - Why do you feel this way? *[if negative, how often would be preferred?]*

- How would you feel about completing the Haywood Arthritis Portal between appointments?
  - Why do you feel this way?
  - What would make this better?

- What different ways do you think the Haywood Arthritis Portal *could* be used to deliver care to people with inflammatory arthritis? (E.g. to triage people on waiting lists)
  - Could you describe any potential positives or negatives to this?

<u>Closing statement</u>

- Do you have any questions or final comments you would like to mention?
- Thank you for participating. If you do have any further questions, please do let us know using the contact details on the participant information form.
