# Supplementary material for: Variation in the feasibility and acceptability of electronic patient-reported outcome measures in patients with inflammatory arthritis
Source: Rheumatol Adv Pract. 2026 Feb 17;10(2):rkag026. doi: 10.1093/rap/rkag026 (PMC13033184; doi:10.1093/rap/rkag026)
Supplement: rkag026_Supplementary_Data [file rkag026_supplementary_data.zip › Supplementary Data S7.pdf]

The Haywood Arthritis Portal Study

Semi-Structured Interview Schedule for Healthcare Professional Participants

<u>Housekeeping</u>

- Welcome and introductions
- Informed consent and consent to record interview
- Check understanding of how to use Microsoft Teams for videocall interviews
- Take breaks as and when needed
- If connection of telephone or videocall fails, interviewer will contact them again
- Collect background information on occupation

Introduction

- You have been invited here because you have viewed electronic patient-reported outcome measures (also called ePROMs) data in at least 20 rheumatology consultations, is that right?
- The reason we are doing this study is to better understand your views on the use of ePROMs in the care of patients with inflammatory arthritis, as well as use of the Haywood Arthritis Portal and Haywood Rheumatology Portal. We would also like to know your thoughts on whether ePROMs and the Haywood Arthritis Portal should be used in the NHS as part of usual patient care.
- Please speak freely as both positive and negative answers are valued. There are no right and wrong answers. If you do not feel comfortable answering any questions, feel free to skip them, or you can choose to stop the interview altogether if you wish.
- Double check if participant is happy to continue with interview.

Section 1: Views on using ePROMs captured by the Haywood Arthritis Portal in the care of patients with inflammatory arthritis

- To begin, can you tell me what your understanding of the Haywood Arthritis Portal is and what you perceive its role to be?

- Can you tell me how you have used ePROMs captured by the Haywood Arthritis Portal?
    - Why? What do you think about this? What would have made the experience better?
    - Can you give me an example of a time when you chose not to use it?

- Aside from the Haywood Arthritis Portal, have you ever used any other methods for capturing PROMS in your clinic? (e.g. paper PROMS, other portal systems like Diamond)
    - Which ones?
    - How do you feel that other ePortal systems compare to the Haywood Arthritis Portal?
        - Better/worse? What did you like/dislike in comparison?

- What difference does the use of the Haywood Arthritis Portal make to the delivery of your consultations?
    - Why do you feel this way?
    - What could be made better to help improve the delivery of your consultations?
    - Do you use the Haywood Arthritis Portal to help guide discussions with patients? Can you tell me more about this?

- How does having ePROMs data available impact patient care?
    - Why do you feel this way? Can you recall any examples?
    - What could be made better? (i.e. to impact patients' arthritis care)

- Generally, how confident do you feel in using ePROMs data via the Haywood Arthritis Portal? (e.g. using the systems, using the patient data in consultations)
    - Can you tell me why you feel that way?
    - What would make you feel more confident?

- What supports or motivates you to use ePROMS data via the Haywood Arthritis Portal (e.g. discussions within rheumatology team)? What further support do you require?

- How much effort does it require for you to use ePROMS data via the Haywood Arthritis Portal?
    - What would have made it easier?

- What drawbacks does the Haywood Arthritis Portal (or ePROMS generally) have?
    - Why do you feel this way?
    - Is there anything you would like to change about ePROMs or the Haywood Arthritis Portal? If so, what? Why?

- Do you think that asking people to complete online questions about their health before their appointment is fair for all people with arthritis?
    - Can you tell me why you feel that way?
    - What could be done to make this fairer?

Section 2: Integration of ePROMs into routine care of patients with inflammatory arthritis

- What are your thoughts about how the Haywood Arthritis Portal/ePROMs can be best used to benefit patients?
    - Why is that?

- Would you be prepared to promote the use of the Haywood Arthritis Portal to patients?
    - Why is that?

- What do you think about using ePROMs in this way as part of your routine practice? Why?
    - How *familiar* does it feel to use the Haywood Arthritis Portal as part of your care?

Closing statement

- Do you have any questions or final comments you would like to mention? Thank you for participating. If you do have any further questions, please do let us know using the contact details on the participant information form.
