# Supplementary material for: Variation in the feasibility and acceptability of electronic patient-reported outcome measures in patients with inflammatory arthritis
Source: Rheumatol Adv Pract. 2026 Feb 17;10(2):rkag026. doi: 10.1093/rap/rkag026 (PMC13033184; doi:10.1093/rap/rkag026)
Supplement: rkag026_Supplementary_Data [file rkag026_supplementary_data.zip › Supplementary Tables.docx]

**Table S1. UK Studies Evaluating the Feasibility and Acceptability of Electronic Patient-Reported Outcome Measures in Routine NHS Care of Patients with Inflammatory Arthritis.**

| **Study (Year)** | **IA Type** | **Study Duration** | **Size, n** | **Female, %** | **Age, years** | **Aim** | **Key Findings** |
| --- | --- | --- | --- | --- | --- | --- | --- |
| Watson  (2024) | RA | 34 months | 171 | 77 | 68% >50 | To evaluate (1) patients and staff experiences, and (2) implementation, including patient and staff acceptability, feasibility, and barriers to and drivers of the implementation of a two-way remote monitoring service (RAID scores collected every 4-weeks) of patients with RA. | Patient engagement was high, and responses were positive about the service. There was 80% agreement across the usability questions, confidence in the service, and patients felt it improved their rheumatology care. Staff views were more mixed and engagement was low. |
| Malley  (2021) | RA, AS | 6 weeks | RA: 149 AS: 26 | RA: 65  AS: 54^ | RA: 64  AS:45^ | To produce a system that allowed integration of patients’ reported data into electronic health records to provide a virtual review of care. | A 62% response rate was achieved. The majority of responders (70%) found it “extremely easy” or “somewhat easy” to complete; remaining responses: “neutral” 20%, “somewhat difficult” 10%, “extremely difficult” 0%. |
| Austin  (2020) | RA | 3 months | 22 | 75 | 32 to 84 | To establish the acceptability and feasibility of collecting daily PGHD using smartphones and integrating PGHD into the electronic health record, using the example of RA. | The 20 self-selected people with RA completed entries on 91% of days over 3-months, and two clinicians found daily ePROMs integrated into electronic health records provided the “bigger picture” of disease activity |

^Sex and age figures provided for responders. IA = Inflammatory Arthritis. n = Number. RAID = Rheumatoid Arthritis Impact of Disease score. AS = Ankylosing Spondylitis. PGHD = Patient-Generated Health Data. PROM = Patient-Reported Outcome Measure.

**Table S2. Characteristics and Digital Access and Skills by HAP Completion Status of Patients Completing the Survey.**

| Characteristic |  | At home (n=172) | In Clinic (n=157) | Non-Completers (n=7) |
| --- | --- | --- | --- | --- |
| *Arthritis Type and Sociodemographic Characteristic* |  |  |  |  |
| IA type, n (%) | RA | 105 (61.0) | 108 (69.7) | 7 (100) |
|  | Peripheral PsA | 22 (12.8) | 10 (6.5) | 0 (0) |
|  | Axial SpA | 28 (16.3) | 24 (15.5) | 0 (0) |
|  | Other* | 17 (9.9) | 13 (8.3) | 0 (0) |
| Age, mean (95% CI) |  | 58.3 (56.3, 60.4) | 61.5 (59.6, 63.5) | 64.4 (50.5, 78.4) |
| Gender, n (%) | Female | 128 (74.4) | 155 (66.5) | 5 (71.4) |
| Ethnicity, n (%) | White | 160 (97.6) | 149 (100) | 7 (100) |
|  | Non-white | 4 (2.4) | 0 (0) | 0 (0) |
| Occupation, n (%) | Employed | 66 (40.2) | 48 (32.7) | 4 (57.1) |
|  | Unemployed | 27 (16.5) | 27 (18.4) | 0 (0) |
|  | Retired | 65 (39.6) | 70 (47.6) | 3 (42.9) |
|  | Other | 6 (3.7) | 2 (1.4) | 0 (0) |
| IMD Quintiles, n (%) | 1 (Most deprived) | 37 (23.0) | 41 (27.9) | 1 (14.3) |
|  | 2 | 20 (12.4) | 25 (17.0) | 2 (28.6) |
|  | 3 | 27 (16.8) | 28 (19.0) | 1 (14.3) |
|  | 4 | 40 (24.8) | 28 (19.0) | 1 (14.3) |
|  | 5 (Least deprived) | 37 (23.0) | 15 (17.0) | 2 (28.6) |
| Low health literacy, n (%) |  | 19 (11.7) | 44 (29.7) | 2 (28.6) |
| *Digital Access and Skills* |  |  |  |  |
| Access to internet, n (%) |  | 162 (98.2) | 128 (85.3) | 5 (71.4) |
| Access to internet on own smartphone, n (%) |  | 143 (87.7) | 93 (69.9) | 4 (57.1) |
| Digital skills, n (%) | Foundation | 139 (84.8) | 105 (71.9) | 3 (60) |
|  | Partial foundation | 25 (15.2) | 27 (18.5) | 0 (0) |
|  | Digitally excluded | 0 (0) | 14 (9.6) | 2 (40) |
| Low eHealth literacy, n (%) |  | 25 (15.3) | 47 (32.2) | 4 (57.1) |

IA = Inflammatory Arthritis. IMD = Indices of Multiple Deprivation. e = Electronic. RA = Rheumatoid Arthritis. SpA = Spondyloarthopathy. *Includes peripheral SpA other than PsA and undifferentiated IA.

**Table S3. Healthcare Professionals’ NoMAD Instrument Item Scores for ePROMs**

| Construct | Item | Response Option | n (%) |
| --- | --- | --- | --- |
| Coherence | I can see how having ePROMs differs from usual ways of working | Agree/strongly agree | 10 (90.9) |
|  |  | Neither agree nor disagree | 1 (9.1) |
|  |  | Disagree/strongly disagree | 0 (0) |
|  | Staff in this organisation have a shared understanding of the purpose of ePROMs | Agree/strongly agree | 7 (63.6) |
|  |  | Neither agree nor disagree | 3 (27.3) |
|  |  | Disagree/strongly disagree | 1 (9.1) |
|  | I understand how ePROMs affects the nature of my own work | Agree/strongly agree | 10 (90.9) |
|  |  | Neither agree nor disagree | 1 (9.1) |
|  |  | Disagree/strongly disagree | 0 (0) |
|  | I can see the potential value of ePROMs for my work | Agree/strongly agree | 10 (90.9) |
|  |  | Neither agree nor disagree | 1 (9.1) |
|  |  | Disagree/strongly disagree | 0 (0) |
| Cognitive Participation | There are key people who drive the use of ePROMs forward at our NHS Trust and get others involved | Agree/strongly agree | 11 (100) |
|  |  | Neither agree nor disagree | 0 (0) |
|  |  | Disagree/strongly disagree | 0 (0) |
|  | I believe that participating in using ePROMs is a legitimate part of my role | Agree/strongly agree | 9 (81.8) |
|  |  | Neither agree nor disagree | 2 (18.2) |
|  |  | Disagree/strongly disagree | 0 (0) |
|  | I’m open to working with colleagues in new ways to use ePROMs | Agree/strongly agree | 11 (100) |
|  |  | Neither agree nor disagree | 0 (0) |
|  |  | Disagree/strongly disagree | 0 (0) |
|  | I will continue to support the use of ePROMs at our NHS trust | Agree/strongly agree | 11 (100) |
|  |  | Neither agree nor disagree | 0 (0) |
|  |  | Disagree/strongly disagree | 0 (0) |
| Collective Action | I can easily integrate using ePROMs into my existing work | Agree/strongly agree | 9 (81.8) |
|  |  | Neither agree nor disagree | 2 (18.2) |
|  |  | Disagree/strongly disagree | 0 (0) |
|  | Using ePROMs disrupts working relationships | Agree/strongly agree | 0 (0) |
|  |  | Neither agree nor disagree | 1 (9.1) |
|  |  | Disagree/strongly disagree | 10 (90.9) |
|  | I have confidence in other people’s ability to use ePROMs | Agree/strongly agree | 2 (18.2) |
|  |  | Neither agree nor disagree | 9 (81.8) |
|  |  | Disagree/strongly disagree | 0 (0) |
|  | Work is assigned to those with skills appropriate to use ePROMs | Agree/strongly agree | 7 (63.6) |
|  |  | Neither agree nor disagree | 4 (36.4) |
|  |  | Disagree/strongly disagree | 0 (0) |
|  | Sufficient training is provided to enable staff to use ePROMs data | Agree/strongly agree | 6 (54.5) |
|  |  | Neither agree nor disagree | 3 (27.3) |
|  |  | Disagree/strongly disagree | 2 (18.2) |
|  | Sufficient resources are available to support ePROMs | Agree/strongly agree | 4 (36.4) |
|  |  | Neither agree nor disagree | 5 (45.5) |
|  |  | Disagree/strongly disagree | 2 (18.2) |
|  | Management adequately supports ePROMs | Agree/strongly agree | 3 (27.3) |
|  |  | Neither agree nor disagree | 6 (54.5) |
|  |  | Disagree/strongly disagree | 2 (18.2) |
| Reflexive Monitoring | I am aware of reports about the effects of ePROMs | Agree/strongly agree | 4 (36.4) |
|  |  | Neither agree nor disagree | 4 (36.4) |
|  |  | Disagree/strongly disagree | 3 (27.3) |
|  | The staff agree that having ePROMs is worthwhile | Agree/strongly agree | 6 (54.5) |
|  |  | Neither agree nor disagree | 4 (36.4) |
|  |  | Disagree/strongly disagree | 1 (9.1) |
|  | I value the effects that ePROMs data has had on my work | Agree/strongly agree | 9 (81.8) |
|  |  | Neither agree nor disagree | 0 (0) |
|  |  | Disagree/strongly disagree | 1 (9.1) |
|  | Feedback about the Haywood Arthritis Portal can be used to improve it in the future | Agree/strongly agree | 11 (100) |
|  |  | Neither agree nor disagree | 0 (0) |
|  |  | Disagree/strongly disagree | 0 (0) |
|  | I can modify how I work with ePROMs data | Agree/strongly agree | 10 (90.9) |
|  |  | Neither agree nor disagree | 1 (9.1) |
|  |  | Disagree/strongly disagree | 0 (0) |

ePROMs = electronic Patient Reported Outcome Measures.

**Table S4. Patient Acceptability of ePROMs in Routine Care by Completion Status**

| TFA Domain |  | Quantitative Findings by Completion Status | | Qualitative Findings | Meta-Inference |
| --- | --- | --- | --- | --- | --- |
|  |  | **Home, n (%)** | **Clinic, n (%)** |  |  |
| Global Acceptability | Acceptable/completely acceptable  No opinion  Unacceptable/completely unacceptable | 162 (94.2)  3 (1.7)  7 (4.1) | 129 (82.7)  16 (10.3)  11 (7.1) | - | - |
| Affective attitude  (Like/dislike ePROMs) | Like/strongly like  No opinion  Dislike/strongly dislike | 100 (58.1)  63 (36.6)  9 (5.2) | 80 (51.3)  61 (39.1)  15 (9.6) | Patients generally liked or felt indifferent towards ePROMs regardless of completion type. Example quote: “*At home it’s more private to do things like that and easier to do and I’d have felt more anxious if I’d have done it sitting while I was waiting to go in to see the consultant”* (patient_1071). | Convergence: slightly more home completers liked/strongly liked ePROMs than clinic completers in quantitative findings, which could be explained by a reported finding that home completion enabled in-advance preparation for appointments, which lessened patients’ anxiety. |
| Ethicality  (Fairness of ePROMs) | Fair/very fair  No opinion  Unfair/very unfair | 132 (76.7)  22 (12.8)  18 (10.5) | 110 (70.5)  30 (19.2)  16 (10.3) | Most thought it was fair to offer all people with inflammatory arthritis the opportunity to complete ePROMs but acknowledged that some people would have challenges undertaking them at home, with a clinic option needed. Example quote: “*Any computer, or phone, or iPads, and things, I think older people certainly struggle with. I think, you know, they – it’d probably be, erm, better for them to just be asked the questions”* (patient_2024). | Divergence: quantitative findings showed both subgroups mostly thought ePROMs were fair/very fair, although home completers reported higher levels of fairness than clinic completers. This differs to qualitative findings, where patients felt home completion might be more challenging for those without internet access or requiring NHS staff support. |
| Perceived effectiveness  (ePROMs likely to help care) | Agree/strongly agree  No opinion  Disagree/strongly disagree | 156 (90.7)  9 (5.2)  7 (4.1) | 135 (86.5)  18 (11.5)  3 (1.9) | Mostly, participants believed ePROMs were likely to help their arthritis care and described development opportunities for further benefits. There were no notable differences between subgroups. Example quote:*“It gives you a longer reference period of recording how you’re feeling, your symptoms, the standard of living, even the mental health side of it. You don’t think there’s anything wrong but the answers you give could flag something up and something could be put in place earlier or if medication is not working for you, you’ve got a way of reporting it”* (patient_1055). | Convergence: quantitative and quantitative findings showed most in both subgroups agreed/strongly agreed ePROMs were likely to help care. |
| Self-efficacy  (Confident answering ePROMs) | Confident/very confident  No opinion  Unconfident/very unconfident | 153 (89.0)  6 (3.5)  13 (7.6) | 128 (82.1)  17 (10.9)  11 (7.1) | Confidence was high in both subgroups despite some uncertainties with answering questions and general technological skills. Example quote: “*Well as long as I’d got my granddaughter with me to start with, I was alright. I could do it myself now you know, but to sort of log it on and that, I can do some things online but other things I need help with”* (patient_5008). | Convergence: both subgroups mostly felt confident/very confident answering ePROMs, with qualitative findings indicating this was due to HAP being user-friendly and clear to understand. Where lack of confidence was described, this was similar across subgroups. For example, two participants (one home completer; one clinic completer) stated they required support to complete ePROMs for the first time, which was provided. |
| Intervention coherence  (Clear how ePROMs help care) | Agree/strongly agree  No opinion  Disagree/strongly disagree | 155 (90.1)  9 (5.2)  8 (4.7) | 132 (84.6)  17 (10.9)  7 (4.5) | Patients generally understood the purpose of ePROMs and how they help care. This was the case for both pre-clinic and in-clinic completers. Example quote: *“I thought it was to save time, that’s really what I assumed it was, to compress those initial questions that are asked when you get in, to get that done so you can focus on actually treatment in the clinic itself. So, I thought it was quite logical”* (patient_2105). | Convergence: most of both home and clinic completers agreed/strongly agreed it was clear how ePROMs help their care. This appears due to many patients envisioning the potential benefits of ePROMs and considering them be a logical addition to their care. |
| Burden  (Effort to complete ePROMs) | No effort/little effort  No opinion  A lot of effort/huge effort | 150 (87.2)  8 (4.7)  14 (8.1) | 128 (83.1)  16 (10.4)  10 (6.5) | Most patients from both subgroups did not find ePROMs burdensome. This was particularly the case for clinic completers who described ePROMs completion as non-disruptive to their leisure time. Example quote: *“Being at the hospital and doing it was better for me, because I was already there, and able to. So, I would prefer to do it that way, than doing – because I’m so busy with other things”* (patient_2024). | Divergence: most considered using ePROMs to be of no effort/little effort in both groups. Although proportionally more home completers reported ePROMs to be less effort than clinic completers, this differs from qualitative findings in which patients described clinic completion to be less burdensome as it reduces boredom whilst waiting for appointments and does not take up their own leisure time. |
| Opportunity costs  (ePROMs interfere with other priorities) | Agree/strongly agree  No opinion  Disagree/strongly disagree | 13 (7.6)  19 (11.0)  140 (81.4) | 18 (11.5)  42 (26.9)  96 (61.5) | Opportunity costs related to ePROMs were mentioned in interviews, however, most participants of both subgroups thought these were minimal. Example quote: *“That’d be fine for me; as long as it didn’t stop me seeing [consultant] once a year and my nurses”* (patient_1092). | Divergence: substantially more home completers disagreed/strongly disagreed that ePROMs interfered with other priorities than clinic completers. The main opportunity cost reported by patients was the possibility of ePROMs to replace face-to-face consultations; patient preference was for ePROMs to supplement routine appointments. However, this did not differ between home and clinic completers. |

**Table S5. Interview Quotes Mapped to TFA Domains**

| TFA Domain | Quotes |
| --- | --- |
| Affective Attitude | *“I can just remember saying to my mum, ‘Oh, I’m ever so glad they’ve asked these questions’”*. (patient_1066) |
| Ethicality | *“Providing they have got access to the internet … But then if they don’t answer them online at home, provided that they’ve got the opportunity to answer them in the clinic, then it’s all good”* (patient 1066).  *“I guess one thing is already being done and that’s giving people the opportunity to complete it (...) in the waiting room before they come in”.* (HCP_4008)  *“there are some clinicians who don’t use it. I think it’s mostly based within particular clinics, but if you do see somebody who has been seen in a different clinic where they haven’t used the portal, in fact this did come up recently where a patient had been seen by a doctor who wasn’t using the portal. Erm, you know, then you haven’t got that ongoing record of how they were doing at that point in time, that sort of more objective record.” (4008)* |
| Perceived Effectiveness | *“It probably makes you think about how you actually are before you get there if you like, to fill the portal in, so you’ve already sort of got the answer to the questions in your head that you might be asked when you get there”.* (patient_2055)  *“I know this sounds daft, but you can be totally honest. You don’t feel like you’re over-exaggerating things because you’re not talking to a person*”. (patient_1066)  *“If you wanted to discuss escalation or de-escalation of care, by having these sorts of facts and figures and a graph in front of you, you can encourage the patient to take better decisions”.* (HCP_4007)  *“I was able to use it as part of the discussion, they would say, ‘oh, I'm getting that, okay, I can continue as I am.’ but then that [ePROMs] could show them well, ‘You've said, you can't get dressed in the morning, your pain’s 10 out of 10, you can't do the things that you want to do.’ And that helps inform that discussion and helps them reflect back, that actually, their arthritis is impacting the day-to-day function. And both the two examples, I'm thinking of, both patients’ kind of accepted the recommendation that it was important to switch their treatment”.* (HCP_4002)  *“I’m a bit of an old-fashioned beast in some ways. You know, I trained in rheumatology a long time ago. I guess I’m maybe slightly sceptical about some of these patient reported outcomes. Again, maybe it’s just familiarity and confidence in the ability to interpret them”.* (HCP_4008)  *“Well, my sort of main incentive really had been to support [clinician] initially (…) But also in terms of patient care, as I have used it I have seen examples of situations where it is undoubtedly useful to be able to show patients the figures to show their progress over time. So, I guess then that generates further motivation in itself”.* (HCP_4008)  *“The answers you give could flag something up and something could be put in place earlier. Or, if medication is not working for you, you’ve got a way of reporting it rather than having to try and get another appointment”.* (patient_1055)  *“If their ePROMs suggest their BASDAI’s over 4 and the pains score’s over 4 and again in consultation with them and discussion with them, actually you’re all in agreement, then that would be an indication that then I would go and speak to one of the consultants and say ‘look we might need to escalate this because of the raised ePROMs score’”.* (HCP_4014) |
| Self-Efficacy | *“I was quite happy with the way it worked and to fill it in and move onto the next page and things like that, yeah I was quite happy doing that”.* (patient_2055)  *“I feel absolute confidence in using them. There are guidelines and treatment decisions that can be made based on these scores very easily”.* (HCP_4007)  *“When they sort of ask you how have you been feeling in the last fortnight, I have to think, ‘well I don’t know really’ (…) I felt that was a little bit difficult to answer in some ways”*. (patient_5008)  *“I think some of the patients, you know, the answers that they've put, don't reflect what they tell you in the clinic”* (HCP_4002)  *“When you’re doing it for the first time you’ve nothing to compare it to. (…) when you have appointment 2 you need to be able to view what you said before so you can think, was it better or worse than it was before?”* (patient_2105) |
| Intervention Coherence | *“I wasn’t really sure actually what it was. Obviously now I do (…) but when I read into it more then I understood what I would have to do”.* (patient_1071)  *“With it being health and obviously data protection and everything, and I knew where it had come from, and I knew what it was all about. No, I had no qualms at all about it”.* (patient_1071)  *“I think it would be helpful to have a more streamlined process for managing some of the issues identified e.g., depression. I am not an expert in this and sometimes I feel uncomfortable asking GPs to deal with things when they are already swamped”* (HCP_4002) |
| Burden and Opportunity Costs | *“I’ve got just as busy a life as the doctors and the nurses. So, if it’s going to make my life simpler and their lives simpler, then I’m quite happy to do it (…) As long as it didn’t stop me seeing [clinician] once a year and my nurses”.* (patient_1092)  *“The main thing I use it for, if it’s recorded, is when I’m calculating a disease activity score. I can enter my clinician-assessed tender and swollen joint count and the blood test (…) combine it with the patient global and therefore that gives me the disease activity score. And obviously if the patient hasn’t recorded it in advance, then I’d have to ask the patient global question. So it avoids me having to do that”.* (HCP_4008)  *“When you go in, you’re having to say exactly what you’ve written and if they’re asking you again, they’ve obviously not read what you’ve written. So, when are they going to read this?”* (patient_1092)  *“I don’t find the medications particularly helpful (…) cos I’ve gotta ask them about their medications anyway”.* (HCP_4008)  *“Maybe the patient, you know, states, they're really low in mood or whatever. And then you’re then duty bound to ask about it, aren't you? So inadvertently, you've increased your consultation time, which is not a problem, because that's, I guess that's about providing good patient care, isn't it?”* (HCP_4002)  *“It times out quite quickly. If you are inputting data on a section, if you get distracted or take too long due to discussion at the same time, it can time out then you have to close it and open it back up again to input the data, but you have to start from scratch”.* (HCP_4014)  *“I think there ought to be some sort of a shortcut in there that if you’ve had a recent appointment you can say ‘yeah as it was 2 weeks ago’ and just fill in, I don’t know, just a smaller section rather than the whole thing each time”.* (patient_2055)  *“When patients are trying to use the tab [tablet] to fill the data because of our healthcare nurse crisis where we barely had one nurse to cover three roles, we’re losing the support of these individuals to help the patients fill these data”.* (HCP_4007)  *“The old-fashioned paper ones – you know, the question then is what you do with that piece of paper… file it into notes or would we have to take a photo of it and scan it in and things like that? So, and then it’s harder to find again”.* (HCP_4013) |
