# Supplementary material for: Variation in the feasibility and acceptability of electronic patient-reported outcome measures in patients with inflammatory arthritis
Source: Rheumatol Adv Pract. 2026 Feb 17;10(2):rkag026. doi: 10.1093/rap/rkag026 (PMC13033184; doi:10.1093/rap/rkag026)
Supplement: rkag026_Supplementary_Data [file rkag026_supplementary_data.zip › Supplementary_Figures.docx]

**Figure S1. General Acceptability of ePROMs by Patient Characteristics for Home Completers**

**
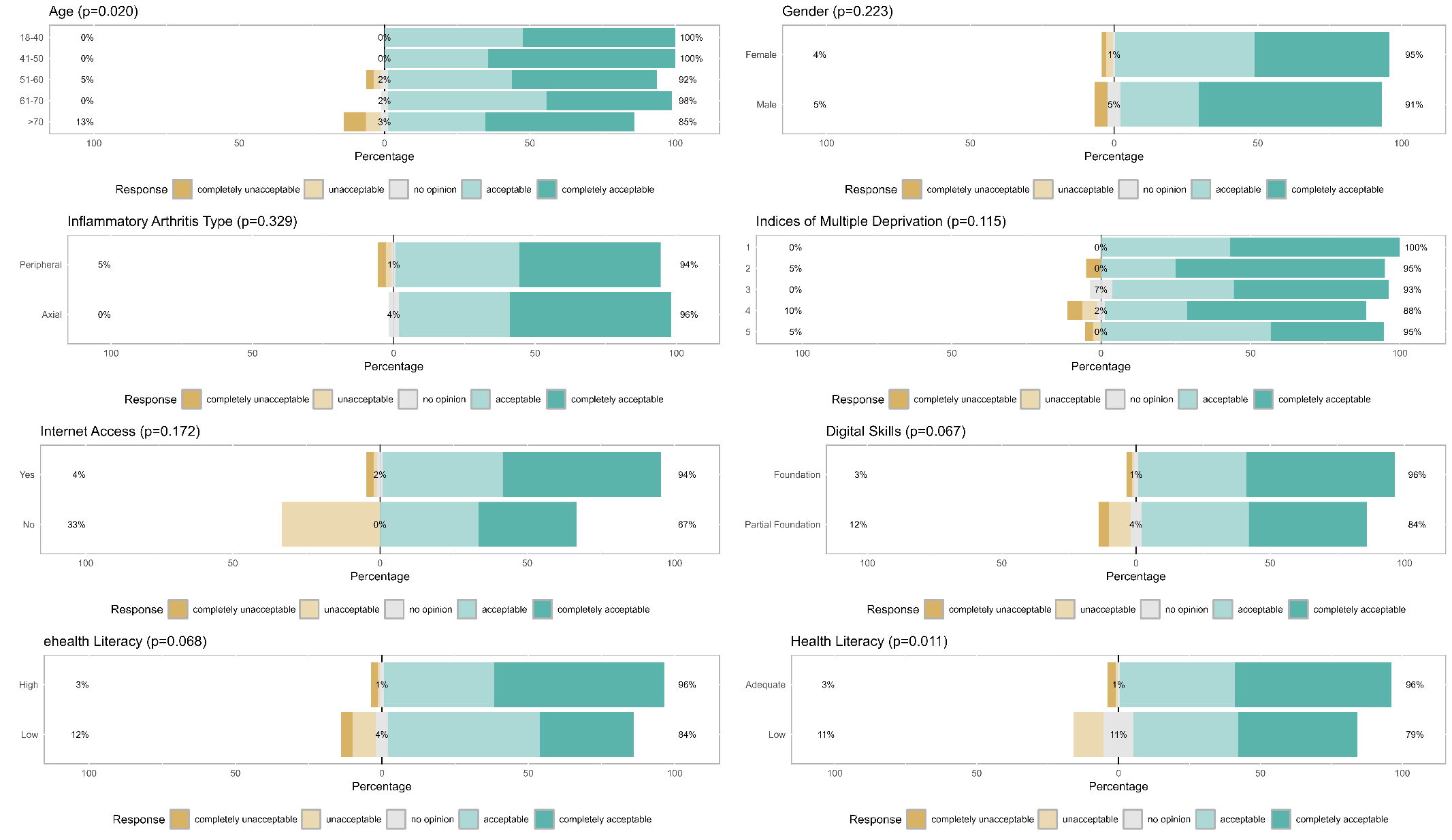
**

**Figure S2. General Acceptability of ePROMs by Patient Characteristics for Clinic Completers**

**
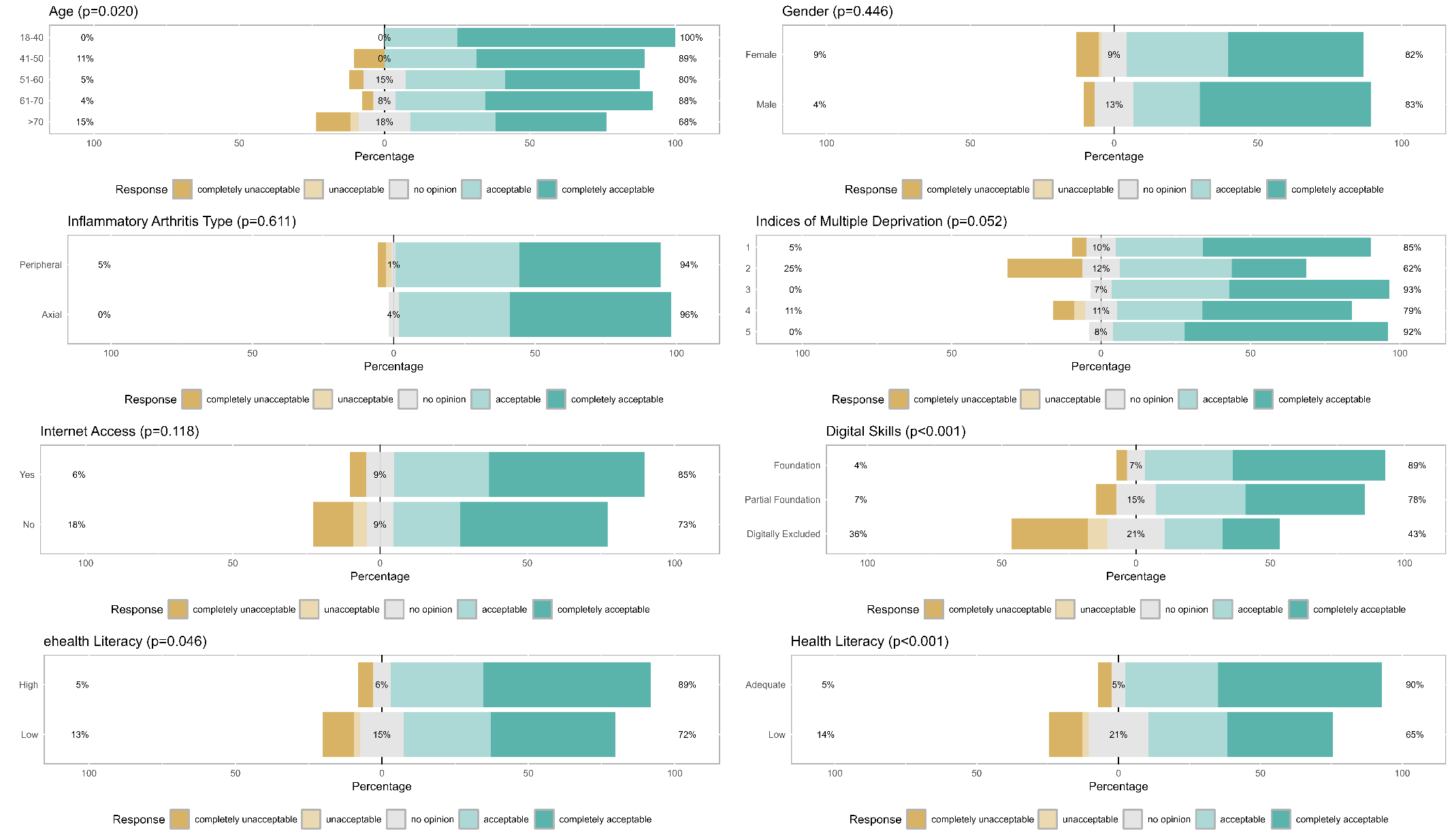
**
